# Maintenance of Tissue Pluripotency by Epigenetic Factors Acting at Multiple Levels

**DOI:** 10.1371/journal.pgen.1005897

**Published:** 2016-02-29

**Authors:** Devendran A. Sadasivam, Der-Hwa Huang

**Affiliations:** 1 Molecular and Cell Biology, Taiwan International Graduate Program, Academia Sinica and Graduate Institute of Life Sciences, National Defense Medical Center, Taipei, Taiwan; 2 Institute of Molecular Biology, Academia Sinica, Taipei, Taiwan; Umeå University, SWEDEN

## Abstract

Pluripotent stem cells often adopt a unique developmental program while retaining certain flexibility. The molecular basis of such properties remains unclear. Using differentiation of pluripotent *Drosophila* imaginal tissues as assays, we examined the contribution of epigenetic factors in ectopic activation of Hox genes. We found that over-expression of Trithorax H3K4 methyltransferase can induce ectopic adult appendages by selectively activating the Hox genes *Ultrabithorax* and *Sex comb reduced* in wing and leg discs, respectively. This tissue-specific inducibility correlates with the presence of paused RNA polymerase II in the promoter-proximal region of these genes. Although the *Antennapedia* promoter is paused in eye-antenna discs, it cannot be induced by Trx without a reduction in histone variants or their chaperones, suggesting additional control by the nucleosomal architecture. Lineage tracing and pulse-chase experiments revealed that the active state of Hox genes is maintained substantially longer in mutants deficient for HIRA, a chaperone for the H3.3 variant. In addition, both HIRA and H3.3 appeared to act cooperatively with the Polycomb group of epigenetic repressors. These results support the involvement of H3.3-mediated nucleosome turnover in restoring the repressed state. We propose a regulatory framework integrating transcriptional pausing, histone modification, nucleosome architecture and turnover for cell lineage maintenance.

## Author Summary

During animal development, the primordia of different body parts undergo a series of transitions in which their developmental potency becomes more restricted. Hox genes encode a family of evolutionarily conserved transcriptional factors that are crucial for choosing different paths during transitions. Thus, the transcriptional status of Hox genes is directly linked to the maintenance and developmental direction of pluripotent tissues. As post-translational methylation of histone H3 is pivotal for transcriptional control, we could activate Hox genes and alter the subsequent development of some pluripotent *Drosophila* imaginal tissues by increasing the level of Trithorax that catalyzes activation-related methylation. However, other imaginal tissues remain refractory unless histone variants or their chaperones that directly affect nucleosome dynamics are simultaneously depleted. By monitoring the duration of Hox expression under these conditions, we found that the active state of Hox genes is substantially prolonged, resulting from effective conversion of promoter-associated paused RNA polymerase II into active transcription. Further analyses indicate that these factors are functionally linked to the Polycomb group of epigenetic factors that bestow long-term repression. Our studies demonstrate that developmental constraints are modulated by factors acting at multiple levels, offering a useful approach to tissue re-programming in regeneration medicine and stem cell research.

## Introduction

During metazoan development, totipotent embryonic cells undergo a series of changes to adopt fixed adult features. Accordingly, the potency of these cells becomes progressively restricted. Intense interest has been focused on deciphering the regulatory molecules and mechanisms responsible for developmental restriction and the practical means of lifting such restrictions [1–5].

*Drosophila* larvae consist of a dozen disc-shaped tissues called imaginal discs that represent progenitors of adult appendages. Transplantation experiments show that they often endure passages of transfer in surrogate hosts without changing developmental fates. However in rare cases, discs may adopt alternative developmental paths and transform into corresponding appendages, revealing their pluripotent nature [6]. Their developmental plasticity is further corroborated by developmental switches resulting from mis-expression of transcriptional factors encoded by Hox genes that act as master developmental regulators [7–10].

Hox genes are mainly regulated by two groups of epigenetic factors categorically called Polycomb group (PcG) of repressors and Trithorax group (trxG) of activators after early embryonic stages. One salient function of PcG and trxG is the ability to trimethylate histone H3 on K27 and K4 (H3K27me3 and H3K4me3) to bestow repressive and active chromatin marks on Hox genes, respectively [11–13]. Although these factors often co-exist in the same cells, the on-off state of Hox genes and underlying chromatin marks are not perturbed through mitotic divisions. How such epigenetic inheritance is stably maintained is not fully understood [13–15].

The Hox genes are tightly regulated at the level of transcription. Recent studies have shown that repressed Hox promoters may contain paused RNA polymerase II (RNA-Pol II) in a region about 50 bp downstream of the transcriptional start site (TSS), with phosphorylation at Ser-5 of the C-terminal domain (CTD) of Rpb1 [16–19]. Genome-wide analyses have revealed a strong correlation between paused RNA-Pol II and PcG repressors and/or H3K27me3 marks [4, 20, 21]. In addition to canonical histones, histone variants have been implicated in transcriptional pausing of Hox genes. Enrichment of H2A.Z, an H2A variant, is widely found in nucleosomes immediately downstream of paused RNA-Pol II [22, 23]. Further, H3.3, a replication-independent H3 variant, is enriched at PcG-binding sites of Hox genes [24–26]. In mouse embryonic cells, depletion of H3.3 results in reduced levels of H3K27me3 at promoters of developmentally regulated genes [27]. However, deposition of H3.3 is also known to establish epigenetic memory of the transcriptionally active state [28].

HIRA, an H3.3 chaperone, is critical for the assembly of the paternal genome after fertilization in *Drosophila* [29] and is required for deposition of histone H3.3 at enhancers of active and poised genes [26, 30–32]. Recently, physical interaction between HIRA and PcG proteins has also been shown [27]. However, HIRA-deficient animals appear to be viable [33, 34], and H3.3 enrichment at telomeric regions and some transcription factor binding sites is HIRA-independent, indicating rather complex functions [35, 36].

Conceptually, maintenance of cell lineages must rely on an intricate interplay between factors acting at multiple levels. The identification of relevant factors and their contributions are imperative to gaining a deep understanding of the nature and potential manipulation of cell lineage maintenance [37, 38]. Here, we used a targeted Trithorax (Trx) expression system to alter developmental fates of several imaginal discs and examined the molecular mechanism employed by cooperating epigenetic factors in this process. We found disc-specific poised states are critical for activation of different Hox genes. In addition, the transiently induced active state may be rapidly obliterated by PcG and HIRA-dependent histone turnover, providing a molecular basis for maintenance of cell lineages.

## Results

### Altered tissue development and Hox expression by Trx

**Fig 1:**
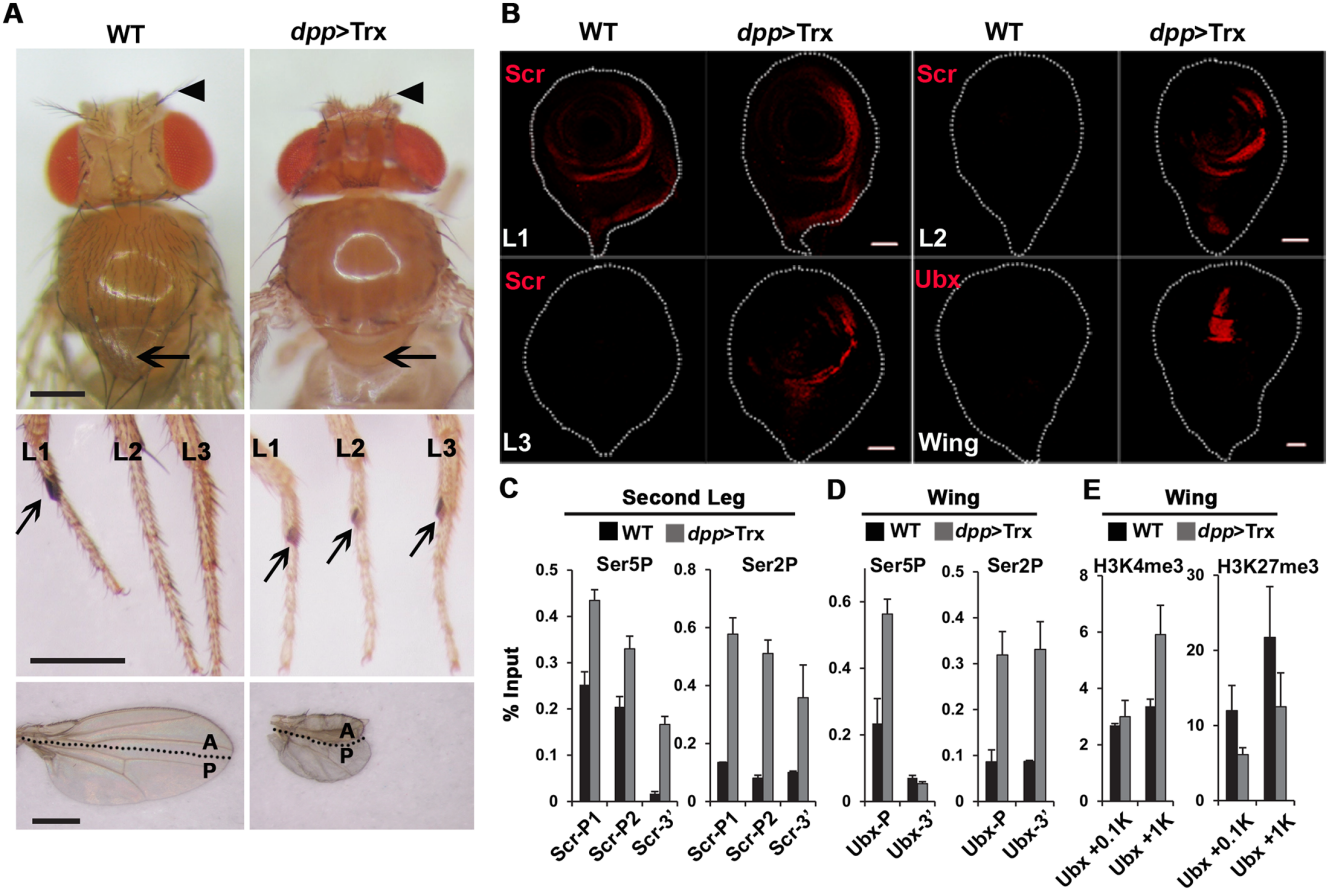
Tissue-specific activation of Hox genes by targeted Trx expression. (A) Aberrant body parts induced by *dpp*-driven Trx (*dpp*>Trx). Top panel, wild-type (WT) adult with normal aristae (arrow head) and notum (arrow), *dpp*>Trx adult lacking aristae and notum. Middle panel, WT adult with sex comb on first leg only (L1, arrow), *dpp*>Trx adult with sex combs on all three legs (L1-L3). Bottom panel, WT adult with normal wings, *dpp*>Trx adult with severely reduced wings. Anterior (A) and posterior (P) compartments of wings and their boundaries are marked. Scale bars in all adult phenotype images represent 0.3 mm. (B) Induction of ectopic Hox proteins by *dpp*>Trx in imaginal discs. Leg and wing discs from third instar larvae were stained with anti-Scr and anti-Ubx antibodies (red), respectively. Ectopic Scr signals in L2/L3 discs and Ubx signals in wing discs are seen in *dpp*>Trx but not in WT samples. Discs are outlined by white dots. Scale bars in all confocal images represent 50 μm. (C-E) Occupancy of RNA polymerase II and tri-methylated histone H3 in Hox genes. Cross-linked chromatin from the second leg (C) and wing discs (D,E) of WT and *dpp*>Trx larvae were immunoprecipitated by different antibodies and quantified by qPCR for promoter-proximal and 3’ regions of different Hox genes. (C,D) Ser5P and Ser2P indicate specific phosphorylated isoforms of RNA-Pol II. (E) H3K4me3 and H3K27me3 indicate specific tri-methylation on histone H3. Bar graphs represent the averages ± SD of two separate ChIP experiments with two independent qPCR reactions. The y-axes represent the percentages of 1% input chromatin. P, promoter region; P1, first promoter; P2, second promoter; 3’, 3’ coding region; +0.1K, 100 bp downstream from TSS; +1K, 1 kb downstream from TSS.

Using the *Gal4/UAS* induction system [39], we examined several Gal4 lines that can drive either ubiquitous (*actin*-Gal4) or selective expression in imaginal discs (e.g. *A9*-, *engrailed*- or *dpp*-Gal4 lines) (S1A Fig). The *dpp*-Gal4 line provides a versatile tool to correlate induction of Hox genes in larval stages with multiple phenotypical changes in adults. This line contains a ~4kb 3’ enhancer (i.e. *blk*) of *dpp* that drives expression primarily in larval imaginal discs [40]. In most imaginal discs, *dpp*-Gal4 is expressed in a strip of cells located along the anterior/posterior (A/P) compartmental boundary. Despite limited induction, Trx driven by *dpp*-Gal4 (henceforth *dpp*>Trx) resulted in profound adult phenotypes, including severely reduced wing size, absence of posterior notum, extra sex combs (ESC) on second and third legs (L2 and L3), and loss-of-arista (Fig 1A). Because these phenotypes resembled homeotic transformations resulting from ectopic expression of Hox genes [41, 42], we began to examine Hox protein expression in imaginal discs.

The Hox gene *Sex comb reduced* (*Scr*) is normally expressed in the anterior compartment of the first leg (L1), but not the L2 or L3 discs, to give rise to the sex comb—a unique morphological marker of L1 in male adults. Consistent with the appearance of ESC on adult L2 and L3, *dpp*>Trx induced strong ectopic Scr signals (Fig 1B). In wing discs where the *Ultrabithorax* (*Ubx*) signal is normally absent, strong Ubx signals were induced in the central pouch region by *dpp*>Trx. Thus, *dpp*>Trx can alter the developmental fate of these discs via selective induction of key Hox genes. However, no ectopic Scr was detected in wing discs by *dpp*>Trx, nor was Ubx detected in L1/L2 discs. Moreover, we did not detect ectopic Scr or Ubx signals in eye-antenna discs. Thus, Trx is not capable of inducing Hox genes uniformly in these discs, indicating unique developmental restriction in different discs. We also noticed that not all Trx-expressing cells within the same disc can induce relevant Hox genes. For example, ubiquitous drivers such as *actin*>Trx could only induce Ubx in the wing pouch region and Scr in small regions of anterior L2/L3 discs (S1A and S1B Fig). Clearly, different parts of a given disc are not equally competent for activation of Hox genes.

Previous studies have shown that RNA-Pol II is paused at the proximal *Ubx* promoter in wing discs [18], raising the possibility of a general link between paused Hox promoters and developmental plasticity in different discs. To explore this possibility, we first examined the phosphorylation states of RNA-Pol II for *Scr* and *Ubx* promoters in leg and wing discs, respectively. Paused RNA-Pol II and actively elongating RNA-Pol II are preferentially phosphorylated at the CTD Ser-5 (Ser5P) and Ser-2 (Ser2P) residues, respectively. Using chromatin immuno-precipitation experiments (ChIP) for isolated discs, we found that the levels of Ser5P are much greater in proximal regions of two separate promoters (P1 and P2) of *Scr* than at 3’ distal region in L2 discs (Fig 1C and gene map in S2 Fig). Similarly, elevated Ser5P signals were observed for P1 and P2 promoters in L3 discs, albeit to a lesser extent (S3A Fig). Further analyses of L2 discs revealed low levels of Ser2P from P1 and P2 to 3’ distal regions, indicating the lack of active elongation. These results support that *Scr* promoters are in a paused state in L2/L3 discs. Under *dpp*>Trx induction, both Ser5P and Ser2P showed significant increases across these regions (Fig 1C). Clearly, paused RNA-Pol II in *Scr* promoters is readily stimulated into active elongation by *dpp*>Trx in L2/L3 discs.

Similar to *Scr*, stronger Ser5P, but not Ser2P, signals were found in the proximal region of the *Ubx* promoter than in the 3’ region in wing discs (Fig 1D). Again, *dpp*>Trx resulted in conversion from the paused state into active elongation with large increases of Ser2P in promoter and 3’ regions. We noted that Ser5P was not increased at the 3’ region of *Ubx* as at the 3’ region of *Scr* in L2 discs, presumably reflecting complete removal of Ser5P for large genes (>70 kb, *Ubx*; ~20 kb, *Scr*; see S2 Fig). We also measured the levels of Ser5P in these regions in L2 discs (where *Ubx* is inactive) and in L3 discs (where *Ubx* is constitutively expressed). Compared to L3 discs, low levels of Ser5P were found in both the promoter and 3’ regions of *Ubx* in L2 discs, suggesting that it is not in the paused state (S3A Fig). Interestingly, no ectopic Ubx signals were induced by *dpp*>Trx in L2 discs. Although both wings and L2 are derived from second thoracic segments, the inducibility of Hox genes by *dpp*>Trx apparently correlates with the paused state of Hox promoters rather than the tissue lineage.

We further examined the state of chromatin modification on *Ubx*. Interestingly, *dpp*>Trx did not cause a significant increase of H3K4me3 in the region immediately downstream (+0.1 kb) of the transcription start site (TSS) (Fig 1E). Instead, a substantial increase of H3K4me3 was observed in the region about 1 kb downstream (+1 kb) of the TSS. In addition, the levels of H3K27me3, a repressive modification, showed substantial decreases in both +0.1 kb and +1 kb regions. We noted that Trx was induced by *dpp*>Trx in small portions (< 20%) of wing and leg discs. Thus, our measurements based on whole discs undoubtedly represent underestimates for various changes. Taking this into consideration, we infer that *dpp*>Trx can induce substantial changes in the relative levels of H3K4me3 and H3K27me3 to facilitate conversion of the paused state to active transcription of Hox genes.

### Hox activation is enhanced by mutations of histone chaperones

We next asked whether other factors contribute to conversion between the paused and active elongation states. Using the wing phenotype induced by *dpp*>Trx or *A9*>Trx, we examined the effect of knockdown of factors involved in transcription, chromatin remodeling and histone chaperones (S1 Table and S4 Fig). As expected for factors that promote transcriptional elongation, co-expression of RNAi for PAF1 or ASF showed dominant suppression of the wing phenotype caused by *dpp*>Trx. Unexpectedly, co-expression of RNAi for negative elongation factors such as components of the NELF (NELF-B, D, E) or DSIF complexes (Spt5) [43, 44] also resulted in rescue of the wing defect. Given that NELF knock-down results in reduction of paused RNA-Pol II and elevated nucleosome occupancy in the promoter-proximal region [45], it is conceivable that these effects undermine the efficacy of induction by *dpp*>Trx. In addition, the wing defect was partially rescued in *nej*^*p*^ mutants, a hypomorphic allele of dCBP coding for a histone acetyltransferase. However, RNAi for *ash1*, a trxG, several positive elongation factors and chromatin remodeling factors failed to show any effect, suggesting that they might be functionally redundant or not critical under such conditions.

Canonical H3 is replaced by H3.3 in actively transcribed genes [46, 47], while H2A.Z is enriched in nucleosomes located immediately downstream of paused RNA-Pol II [23]. To examine whether they are involved in Hox regulation, we tested their interactions with *Polycomb* (*Pc*), an epigenetic repressor of Hox genes. Surprisingly, strong genetic interactions were observed (Table 1 and S5 Fig). The average number of extra sex comb teeth (ESCT) on L2/L3 of *Pc*^*4*^ heterozygotes was substantially enhanced from 0.8 to 3.7 by an *H3*.*3B*^*0*^ mutation and from 1.4 to 5.4 by an *H2Av*^*810*^ mutation. These results suggest that both H3.3 and H2A.Z are negatively involved in Hox gene regulation.

**Table 1:** Enhanced ESC phenotype by histone variant and chaperone mutations.

Mutant	*Pc*^*-*^/Bal	*Pc*^*-*^*/*mutant
*Hira*^*ssm*^	0.6 ± 0.1 (120)	4.4 ± 0.2 (120)
*Hira*^*HR1*^	0.7 ± 0.1 (120)	3.4 ± 0.2 (120)
*His3*.*3B*^*0*^	0.8 ± 0.1 (120)	3.7 ± 0.2 (120)
*His3*.*3A*^*2X1*^	1.2 ± 0.2 (120)	1.4 ± 0.1 (120)
*dom*^*3*^	2.4 ± 0.2 (120)	7.6 ± 0.2 (120)
*dom*^*9*^	2.7 ± 0.2 (120)	4.3 ± 0.2 (120)
*H2Av*^*810*^	1.4 ± 0.2 (120)	5.4 ± 0.2 (120)

Females carrying mutations for histone variants or chaperones were crossed to *Pc*^*4*^ males. The numbers of extra sex comb teeth (ESCT) on both L2 and L3 of their progenies were scored. The averages and standard error of the mean (SEM) of ESCT per leg are shown. Total numbers of legs examined are indicated in parentheses. See S5 Fig for the range of % ESCT. Note that none of these histone chaperone or variant mutants show ESCT phenotype alone. Bal, Balancer.

We further examined the roles of HIRA, a H3.3 chaperone, and Domino (*dom*), a H2A.Z chaperone. Although *Hira* or *dom* mutants alone did not show ESCT phenotype in L2/L3 (S6A Fig), they significantly enhanced the ESCT phenotype of *Pc*^*4*^ mutants [48]. The average ESCT increased from ~0.6 to ~4.4 in the *Hira* background, and from ~2.4 to ~7.6 in the *dom* background (Table 1). Therefore, like the corresponding histone variants, these chaperones act as negative regulators of Hox genes. Reciprocal crosses also showed enhanced ESCT when *Pc*^*4*^ and other mutants were combined (S2 Table). Due to their stronger effects, we chose *Hira*^*ssm*^ and *dom*^*3*^ for further studies.

**Fig 2:**
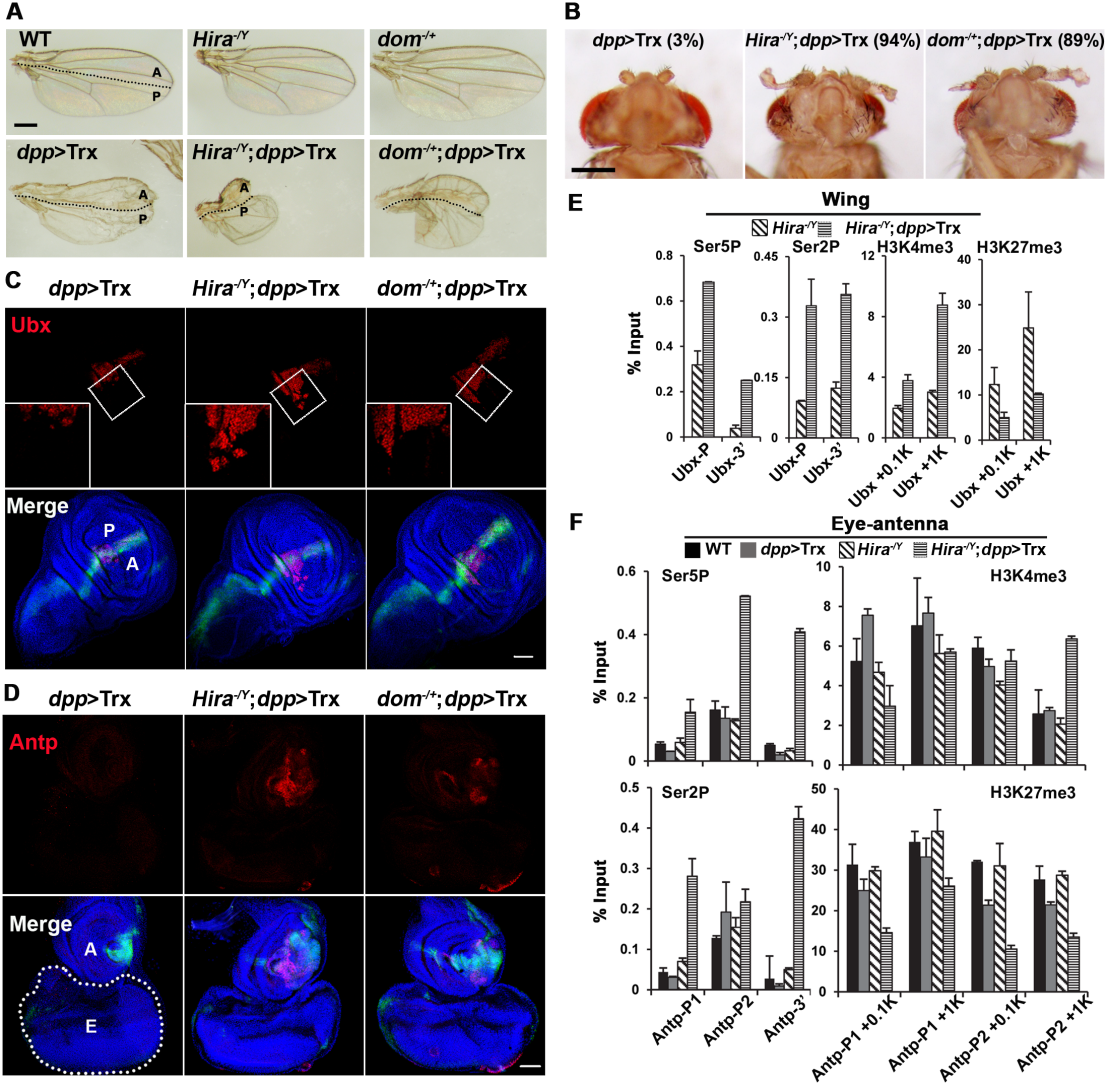
Enhancement of Trx-induced phenotypes in *Hira* and *dom* mutants. (A) Wing phenotypes induced by *dpp*>Trx are enhanced in *Hira* hemizygotes or *dom* heterozygotes. (B) Arista phenotypes induced by *dpp*>Trx are enhanced in *Hira* hemizygotes or *dom* heterozygotes. The percentages of arista-to-leg transformations are indicated in parentheses. Only thin leg-like tissues are induced by *dpp*>Trx alone, while authentic leg tissues are seen in *Hira* or *dom* mutants. (C) Ectopic *Ubx* expression induced by *dpp*>Trx in wing discs is enhanced in *Hira* or *dom* mutants. Top panel, Ubx staining in wing discs (red). Based on GFP signals, the same disc regions are boxed. Enlarged (2.1X) images of boxed regions are shown as inserts. Lower panel, merged images of DNA (blue), Ubx staining (red) with A/P boundaries marked by *dpp*>GFP signals (green). (D) Ectopic *Antp* expression induced by *dpp*>Trx in eye-antenna discs is enhanced in *Hira* or *dom* mutants. Top panel, Antp staining in discs (red). No signal is detectable in the WT sample. Lower panel, merged images of DNA staining (blue), Antp staining (red) and *dpp*>GFP signals (green). Antenna and eye discs are marked by A and E, respectively. The outline of the eye disc is marked by white dots. (A-D) Experiments were carried out at 21°C. (E, F) Occupancy of RNA polymerase II and tri-methylated histone H3 in Hox genes. ChIP analyses were carried out as described in Fig 1C for chromatin samples prepared from wing (E) or eye-antenna (F) discs of different genotypes.

As expected, *dpp*>Trx-induced phenotypes became more pronounced in *Hira* or *dom* mutant backgrounds (Fig 2A). Adult wings from both *Hira*^-/Y^;*dpp*>Trx and *dom*^-/+^;*dpp*>Trx flies showed more severe size reductions than *dpp*>Trx alone. It is noteworthy that the anterior parts of wings were more severely affected than the posterior parts. Similar to the lack of ESCT phenotype, *Hira* and *dom* mutants did not show any wing defect (Fig 2A). However, when combined with overexpression of Trx, *Hira* and *dom* mutants increased the average ESCT for L2/L3 from 2.0/1.6 to 8.2/7.4 and to 6.1/5.2 in *Hira* and *dom* mutants, respectively (Table 2 and S6B Fig). More strikingly, about 90% of *Hira* or *dom* mutants showed strong arista-to-leg transformations, whereas only ~3% weak transformation with thin leg-like tissues was seen in the *dpp*>Trx line alone (Fig 2B).

**Table 2:** Genetic interactions between *dpp*>Trx and chaperones.

	2^nd^ Leg	3^rd^ Leg
*dpp*>Trx	2.0 ± 0.1 (135)	1.6 ± 0.1 (131)
*Hira*^-/Y^;*dpp*>Trx	8.2 ± 0.1 (109)	7.4 ± 0.1 (102)
*dom*^-/+^;*dpp*>Trx	6.1 ± 0.1 (113)	5.2 ± 0.1 (98)

*UAS*-*Trx* females with or without chaperone mutations were crossed to *dpp*-*Gal4* males. The ESCT on L2 and L3 were separately scored. The average and SEM are shown as described previously. Experiments were carried out at 21°C, which generally produced lower ESCT. See S6D Fig for distributions of % ESCT.

Consistent with more severe phenotypes in mutants, strong Ubx and Scr signals were detected in wider regions in wing and leg discs, respectively (Figs 2C and S6C). Using *dpp*>GFP to mark the A/P boundary, we found that ectopic Ubx signals extended beyond the A/P boundary in mutant discs, with more signals in the anterior compartment. These asymmetric patterns correlated with more severe deformation of anterior wings in these mutants. We also examined Hox gene expression in eye-antenna discs. Similar to *dpp*>Trx, there was little if any Ubx or Scr signals in mutant discs. However, strong *Antennapedia* (*Antp*) signals were detected in the region of antennal tissues corresponding to adult arista, providing a direct molecular basis for the arista-to-leg transformation in mutant adults (Fig 2D). Therefore, these chaperones and histone variants both appear to restrict the ability of Trx to activate Hox genes in tissue-specific manners.

We next examined the status of RNA-Pol II occupancy and histone H3 tri-methylation in *Ubx* and *Antp* genes in *Hira* mutant backgrounds. Both wing and eye-antenna discs were collected independently from *Hira*^-/Y^ and *Hira*^-/Y^;*dpp*>Trx larvae and subjected to ChIP analyses. For the *Ubx* gene in wing discs, significant increases of Ser5P and Ser2P were induced by *dpp*>Trx in both promoter and 3’ regions (Fig 2E). These increases were accompanied by increases in H3K4me3 and decreases in H3K27me3 at the +0.1 kb and +1 kb regions. By and large, *dpp*>Trx induces similar changes in both WT and *Hira* backgrounds. However, we noted that either increases in H3K4me3 or decreases in H3K27me3 were more pronounced in *Hira* mutants, particularly in the downstream region.

In WT eye-antenna discs, the P2, but not P1, promoter region of the *Antp* gene showed relatively high levels of Ser5P and Ser2P (Fig 2F). However, only low levels of Ser5P and Ser2P were detected in the 3’ region, indicating a lack of active transcription. Thus, the P2 of *Antp* is poised in the paused state. Surprisingly, *dpp*>Trx failed to induce substantial increases of Ser5P or Ser2P in P1, P2 and downstream regions unless HIRA was inactivated. Thus, unlike *Ubx* in wing discs and *Scr* in L2/L3 discs, *Antp* induction by *dpp*>Trx is strictly controlled by HIRA in eye-antenna discs. Interestingly, *Hira* mutation alone was insufficient to cause any significant change in Ser5P or Ser2P of *Antp* promoters, suggesting that HIRA is not directly involved in modulation of RNA-Pol II. In addition, only low levels of Ser5P and Ser2P were detected in *Scr* and *Ubx* promoters under various genetic backgrounds (S3B Fig). These results further strengthen our earlier observation of a close relationship between inducibility and the paused state for Hox genes.

We further examined H3 tri-methylation on *Antp* in eye-antenna discs. In agreement with the lack of effects on RNA-Pol II occupancy, *dpp*>Trx alone failed to stimulate the level of H3K4me3 in +0.1 kb and +1 kb regions of both P1 and P2 promoters. In *Hira* mutants, however, a selective increase of H3K4me3 was detected in the +1 kb region of P2, but not other regions. In contrast, more uniform reductions were found for H3K27me3, albeit to different extents, in these regions. Thus the ability of *dpp*>Trx to alter the status of H3 tri-methylation is also restricted by HIRA activity in eye-antenna discs.

### Lineage tracing of Hox activation

**Fig 3:**
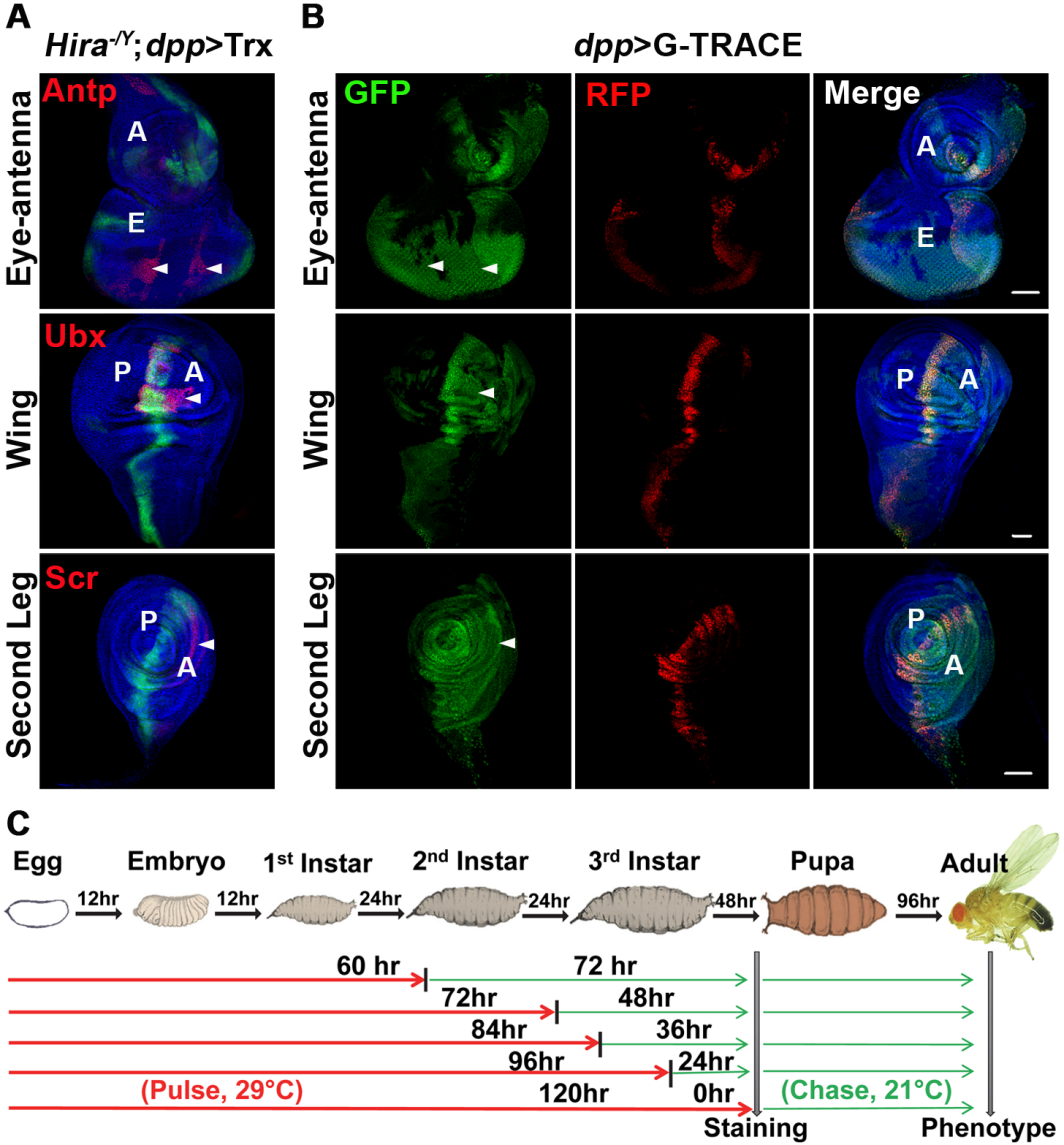
Lineage analyses of Hox-expressing cells that lack *dpp*>GFP. (A) Incompatible Hox and GFP patterns in *Hira* mutants. Staining of Antp, Ubx and Scr in eye-antenna, wing and L2 leg discs, respectively, is shown in merged images with Hox staining (red), DNA staining (blue) and *dpp*>GFP signals (green). Regions showing Hox signals that do not match with *dpp*>GFP signals are indicated by arrowheads (*Hira*-dependent signal). Note that deviant Scr signals are also seen in the *dpp*>Trx background. (B) Comparison of cells with *dpp*-Gal4 activities at different stages. A G-TRACE strategy [49] was used to simultaneously mark cells with *dpp*-Gal4 activities throughout development by a FLP-dependent *Ubi*-GFP and cells with concurrent *dpp*-Gal4 activities by *UAS*-*RFP* in different discs (details in S7A Fig). Merged images of GFP (green), RFP (red) and DNA (blue) are shown. Arrowheads indicate regions with incompatible Hox expression in Fig 3A. (C) Protocol for pulse-chase experiments. A G-TRACE strategy was used for animals carrying a temperature-sensitive Gal80 (*tub*-*Gal80*^*ts*^). Embryos were raised at non-permissive temperature (29°C) for indicated periods (red line) until larval stages to allow cell marking by both *Ubi*-GFP and *UAS*-*RFP*. Subsequently, larvae were returned to permissive temperature (21°C, green line) to block Gal4 activity. The duration for 21°C was roughly estimated based on that for 29°C. The approximate developmental stages are indicated.

In addition to the Antp signals in antennal discs of *Hira* mutants, we noticed strong Antp signals in eye discs that are distant from the region that is under *dpp*-Gal4 induction, as marked by GFP signals (Fig 3A). Similarly, the ectopic Scr signals in L2/L3 discs were located in anterior regions that are distant from *dpp*>GFP in both WT and *Hira* backgrounds. Incompatible GFP and Hox patterns were also observed, albeit to a lesser extent, in anterior wing discs for Ubx. These Hox signals are hereafter referred to as *Hira*-dependent signals. Given the synergistic interactions between *Hira* and *Pc*—a key factor involved in epigenetic memory—we suspected that *Hira*-dependent signals might represent sustained Hox expression from earlier developmental stages. To test this possibility, we first traced the lineage of cells with active *dpp* enhancer at different stages. We exploited a lineage tracing strategy (G-TRACE) to mark cells with *Ubi*>GFP by removing FRT-flanked transcriptional termination signals residing in the 5’-UTR of *Ubi*>GFP via inducible *UAS-FLP* (S7A Fig) [49]. All descendent cells from these marked cells thus become continuously marked by *Ubi*>GFP. Additionally, cells with active *dpp* enhancer near the time of dissection were marked with RFP. Superimposition of these images enabled us to identify the region with active *dpp* enhancer at previous stages. Comparison of the location of Hox signals with superimposed images revealed that *Hira*-dependent signals clearly reside in cells with earlier lineages (Fig 3B).

To further trace the developmental origin of these cells, we performed chase experiments with the introduction of a temperature sensitive Gal80 (Gal80^ts^) into G-TRACE lines to block Gal4 activity at defined stages. Animals were first raised at a non-permissive temperature of Gal80^ts^ (29°C) to allow simultaneous induction of RFP- and FLP-dependent GFP, then shifted to a permissive temperature (21°C) at different time-points to block further expression of RFP and FLP until dissection at the third instar (Fig 3C). Comparison of the GFP- and RFP-marked regions from different time-points enabled us to map the region where *dpp* is temporarily active during different periods. As exemplified for leg discs (S7B Fig), RFP signals induced with either no chase or a 24-h chase were mostly located in regions that corresponded to the A/P boundary. Very little RFP signal was seen in the anterior part of the disc where *Hira*-dependent signals were detected. Conversely, strong GFP signals were observed in this region 48 h before dissection, strongly suggesting that *Hira*-dependent signals were induced by *dpp* activities from earlier stages. Indeed, *dpp* activities from these stages were sufficient to induce Scr signals and leg transformation (see below). Similarly, *dpp* activities were also evident from early stages in regions corresponding to *Hira*-dependent Ubx and Antp signals in wing and eye-antenna discs, respectively (S7B Fig).

### Sustainable Hox expression in *Hira* mutants

**Fig 4:**
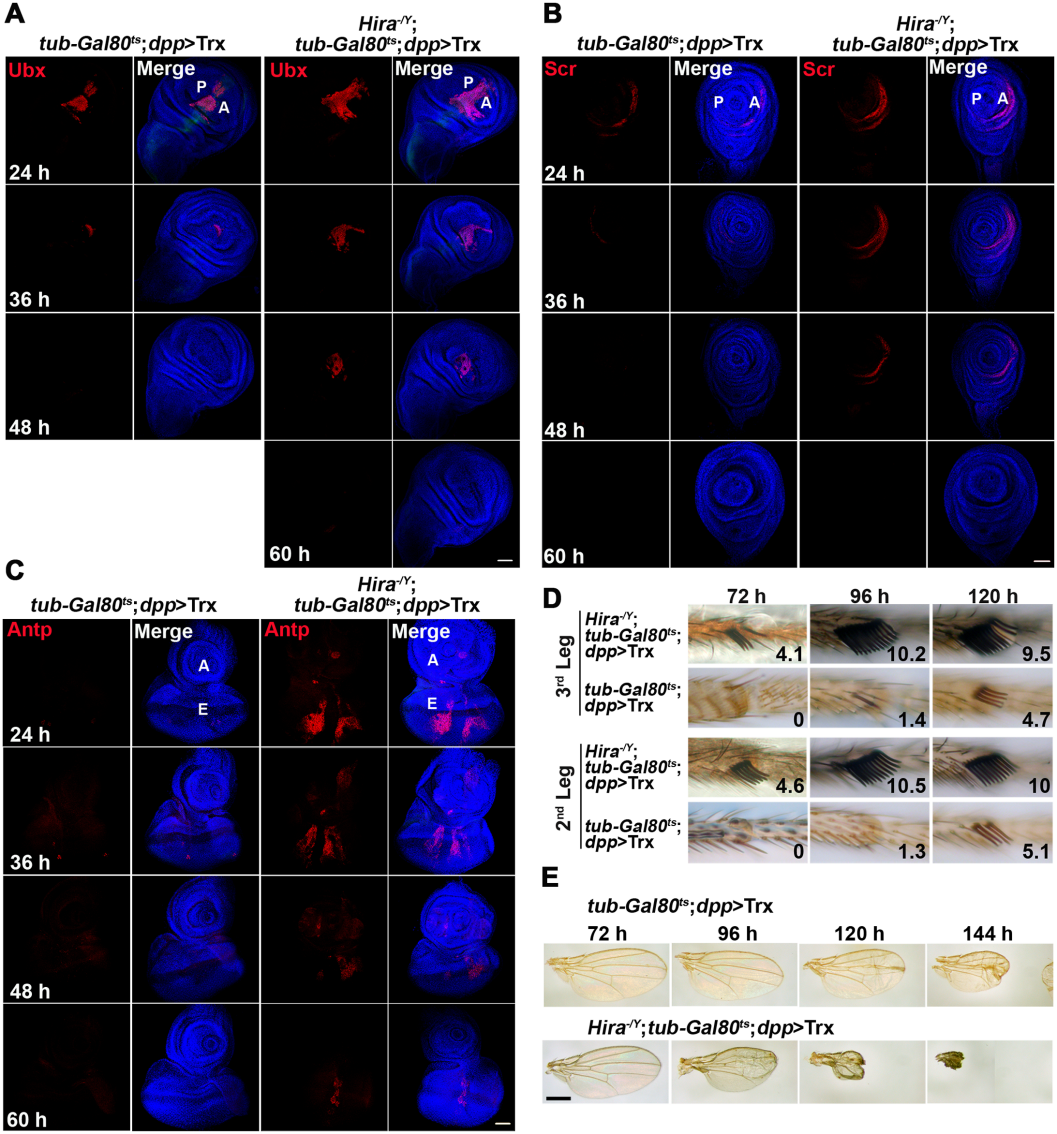
Maintenance of Hox expression in *Hira* mutants. (A-C) Sustained Hox expression in *Hira* mutants. Pulse-chase experiments were performed with different chase periods as indicated (see Fig 3C). Individual staining of Ubx (A), Scr (B) and Antp (C) is shown for wing, L2 leg and eye-antenna discs, respectively. Merged images of Hox (red), DNA (blue) and GFP (green) are also shown. Note that only weak GFP signals can be seen after a 24-h chase. Compared to WT, Hox signals can be detected after longer chases in *Hira* mutants. (D, E) Enhanced adult phenotypes in *Hira* mutants. A similar pulse-chase protocol was adopted except that the chase was extended to eclosion. Hours indicate the duration of pulses. (D) Enhanced ESC phenotypes. The average numbers of sex comb teeth on L2 and L3 are indicated on the top. (E) Enhanced wing phenotypes in *Hira* mutants.

To ascertain the efficacy of *dpp*>Trx activity at early developmental stages, we examined Hox expression in animals treated under similar pulse-chase conditions (Fig 4A and 4B). Substantial amounts of Ubx and Scr signals could still be detected in WT discs after a 24-h chase, whereas very weak or no signals were seen after longer chases (36 h), indicating clearly that the effect of *dpp*>Trx activity during embryonic and early larval stages can’t sustain. However, Ubx and Scr signals were seen even after a 48-h chase in *Hira* mutants. Importantly, these signals appeared to match spatially with the *Hira*-dependent signals described earlier. Thus, both Ubx and Scr could be induced at earlier stages in *Hira* mutants. Remarkably, such induction was sustained for at least 48 h without continuous supply of Trx. A more striking contrast was observed in eye-antenna discs. In the WT background, *dpp*>Trx failed to induce any Antp signals. In contrast, a substantial amount of Antp signal was induced in mutant discs and they remained detectable after a 60-h chase (Fig 4C).

Next, we examined the adult phenotypes caused by these treatments. Although no obvious effect was found in adults treated with a 48-h pulse, extended treatment for 72 h resulted in appearance of ESCT on both L2 and L3 in mutants (avg. ESCT 4.6 and 4.1 for L2 and L3, respectively) (Table 3 and Fig 4D), but not in WT flies. Remarkably, ESCT on L2 and L3 of mutant flies produced by a 96-h pulse were more pronounced than WT flies under the same treatment (10.5 and 10.2 vs 1.3 and 1.4 for L2 and L3, respectively) or flies with continuous *dpp*>Trx throughout development (avg. ESCT ~8.1), and almost approached sex comb tooth number for L1 (10~12). The induction of almost complete transformation of posterior legs by the pulse clearly illustrates the developmental impact of sustainable Scr expression.

**Table 3:** ESC phenotypes induced by *dpp*>Trx pulses.

Trx Induced	2^nd^ leg		3^rd^ leg	
	*dpp*>Trx	*Hira*^-/Y^; *dpp*>Trx	*dpp*>Trx	*Hira*^-/Y^; *dpp*>Trx
48 h	0 (80)	0.8 (79)	0 (82)	0 (83)
72 h	0 (89)	4.6 ± 0.1 (87)	0 (81)	4.1 ± 0.1 (82)
96 h	1.3 ± 0.1 (85)	10.5 ± 0.1 (82)	1.4 ± 0.1 (84)	10.2 ± 0.1 (81)
120 h	5.1 ± 0.1 (84)	10 ± 0.1 (80)	4.7 ± 0.1 (86)	9.5 ± 0.2 (83)
148 h	6.1 ± 0.2 (71)	9.1 ± 0.2 (48)	6.2 ± 0.1 (76)	8.6 ± 0.2 (47)

Females carrying *UAS-Trx* with or without *Hira* mutations were crossed to *tub-Gal80*^*ts*^*;dpp*-*Gal4* males according to the protocol described in Fig 3C. ESCT phenotype was scored as described previously. See S8 Fig for distributions of % ESCT.

Reductions in wing size began to appear in mutant flies treated with a 96-h pulse. Unlike the ESCT phenotype, wing phenotypes continued to deteriorate with longer pulses (Fig 4E). Similarly, a more pronounced arista-to-leg phenotype was also found in mutants with extended pulse period (S3 Table). The frequency of arista-to-leg transformations increased from ~3% to ~40% by prolonging induction from 96 to 168-h. Clearly, the effective period of phenotype induction for arista and wing discs is different from that of leg discs. Such differences probably reflect unique developmental properties of these discs.

### Possible causes for sustainable Hox expression

**Fig 5:**
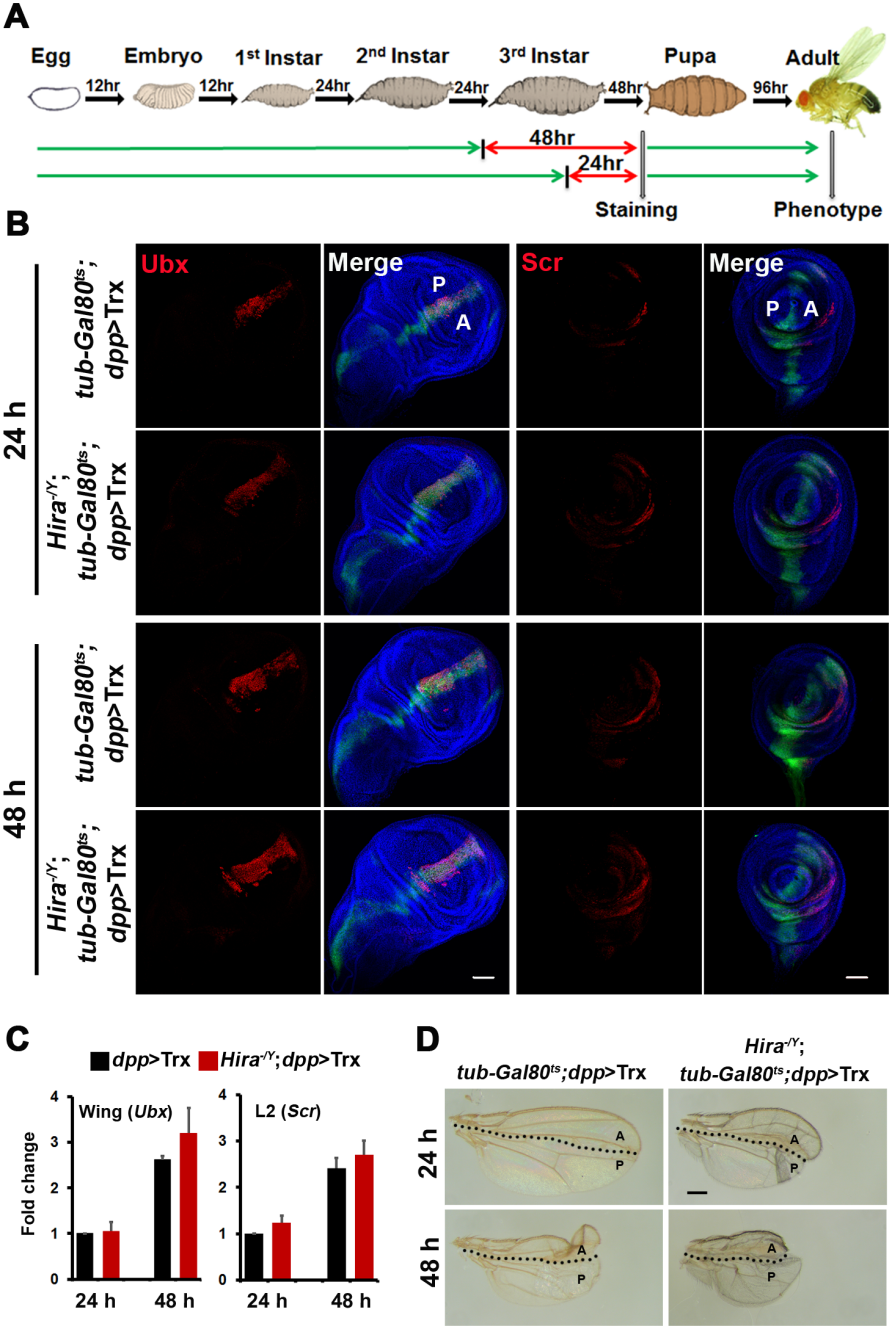
Efficacy of short *dpp*>Trx pulses. (A) Protocol for pulse experiments. Animals carrying *tub-Gal80*^*ts*^;*dpp*>*Trx* were raised at 21°C until late second instar or early third instar (green line) and shifted to 29°C for 24 h or 48 h (red line). These larvae were either dissected or returned to 21°C until eclosion. (B) Induction of Hox expression by short *dpp*>Trx pulses. After 24 or 48 h of *dpp*>Trx pulses, wing and L2 leg discs were stained for Ubx and Scr (red), respectively. For each pulse, comparable levels of Hox expression were induced in WT and *Hira* mutants. (C) Quantitative RNA measurement. Reverse transcription and qPCR were performed for RNA samples isolated from wing and L2 discs. Relative mRNA levels are shown after being normalized to corresponding Rpl32 mRNA expression. Means ± SD from two separate experiments with triplicate are shown. (D) Enhanced wing phenotypes by short *dpp*>Trx pulses in *Hira* mutants. Malformed adult wings induced by 24 h or 48 h *dpp*>Trx pulses are shown for WT and *Hira* mutants.

The stronger effects observed in *Hira* mutants might result from several possibilities. For instance, much more Hox signals might be induced in *Hira* mutants. To examine this possibility, we compared the levels of Ubx and Scr induced by short pulses of *dpp*>Trx in WT and mutant discs. Larvae at early or late second instar were shifted from 21°C to 29°C to inactivate Gal80^ts^ for 24 or 48 h until dissection (Fig 5A). In both cases, we observed comparable levels of Ubx and Scr signals in WT and mutant discs by immunostaining and quantitative RNA measurement (Fig 5B and 5C). When these larvae were shifted back to 21°C to complete morphogenesis, emerging adults showed marked differences in wing and leg phenotypes. Mutant larvae receiving either 24 h or 48 h pulses showed more severe reductions in wing size (Fig 5D). Similarly, ESCT numbers on L2 and L3 of mutants was significantly greater than those of WT adults (Table 4). These results strongly argue against the possibility that *Hira* mutation might directly affect the level of Hox induction. Instead, they suggest that higher levels of Hox signals and stronger phenotypes in *Hira* mutants may be attributed to cumulative effects of sustainable Hox expression during development.

**Table 4:** ESC phenotypes induced by short *dpp*>Trx pulses.

Trx Induced	2^nd^ leg		3^rd^ leg	
	*dpp*>Trx	*Hira*^-/Y^; *dpp*>Trx	*dpp*>Trx	*Hira*^-/Y^; *dpp*>Trx
24 h	0.5 ± 0.1 (50)	2.0 ± 0.2 (50)	0.2 ± 0.1 (50)	1.0 ± 0.1 (50)
48 h	4.2 ± 0.1 (50)	6.0 ± 0.3 (50)	3.2 ± 0.2 (50)	6.0 ± 0.2 (50)

Experiments were done as in Table 3 except that short *dpp*>Trx pulses were given (Fig 5A). See S9 Fig for distributions of % ESCT.

It is noteworthy that although there were only modest increases in Scr signals from the 24 to 48-h treatment (Fig 5B), ESCT showed much greater increases. For example, ESCT number for L2 increased from 2.0 to 6.0 in *Hira* mutants and from 0.5 to 4.2 in WT animals (Table 4). These abrupt increases strongly suggest that the period between 24 and 48 h prior to dissection is critical for the developmental switch of posterior legs, further supporting our earlier observation that continuous *dpp*>Trx is not necessary for extensive leg transformation (Fig 4D).

Next, we examined whether sustainable Hox expression could result from the persistence of Hox or Trx proteins. The rapid decay of simultaneously-induced GFP signals argues against this possibility. To seek direct evidence, we used pulse-chase experiments to examine the persistence of Antp and Scr, which showed more prominent *Hira*-dependent signals, and Trx. These proteins were induced directly from *UAS* lines by *dpp*>Gal4 in WT or *Hira* mutant backgrounds at 29°C and then chased for various periods at 21°C (Fig 3C). In the case of Scr, induced signals disappeared almost completely in L2 discs within a 12-h chase (S10A Fig). Little, if any, Antp signal was detected in eye-antenna discs following an 18-h chase (S10B Fig). In both disc types, induced Trx signals became almost indistinguishable from endogenous Trx following a 6-h chase (S11 Fig). Clearly, the Hox signals observed after a 48-h chase cannot be attributed to the persistence of these proteins.

### Interaction between Hira and PcG

**Fig 6:**
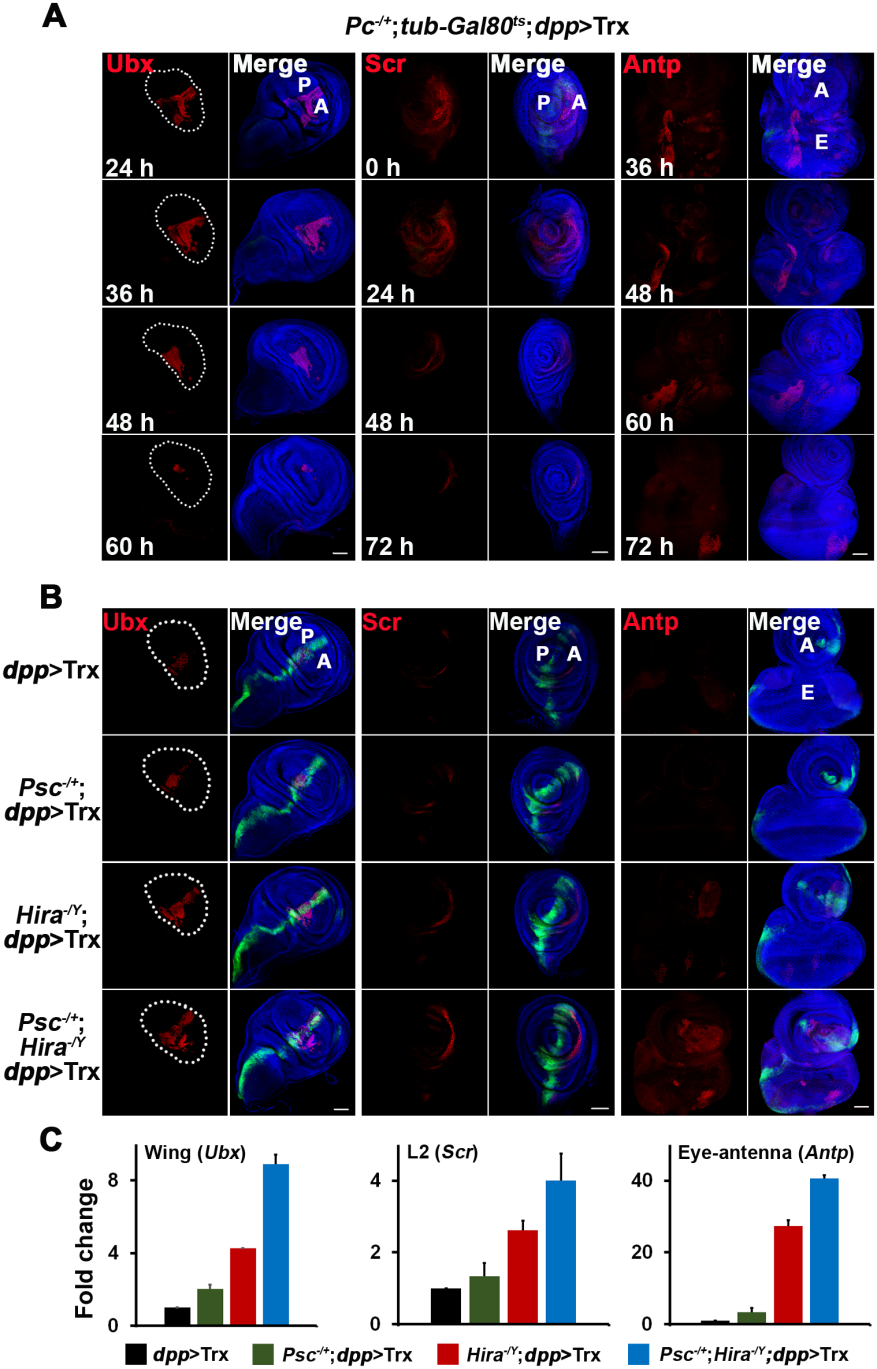
Cooperativity between HIRA and PcG. (A) Sustained Hox expression in *Pc* mutants. The protocol described in Fig 3C was used for pulse-chase experiments in *Pc*^*4*^ mutants. Hours indicate the duration of chases before dissection. The staining of Ubx, Scr and Antp in wing, L2 leg and eye-antenna is shown, respectively. Ubx signals are detectable after a 60-h chase, whereas Scr and Antp signals can still be seen after 72 h chases. Wing pouch is outlined by dots. (B) Enhanced Hox expression in *Hira* and *Psc* double mutants. Merged images of GFP (green), Hox (red) and DNA (blue) are shown. Hox expression induced by *dpp*>Trx in the following genotypes is compared: WT, *Psc*^*1*^, *Hira*^*ssm*^, and *Hira*^*ssm*^;*Psc*^*1*^. (C) Enhanced RNA levels in *Hira* and *Psc* double mutants. RT and qPCR analyses were carried out as described in Fig 5C for Ubx, Scr and Antp in wing, L2 and eye-antenna discs, respectively. (B, C) Experiments were carried out at 21°C.

Our observation that *Hira* mutation could enhance ESCT phenotypes of the *Pc* mutation suggested that the sustainable Hox expression in *Hira* mutants might be mechanistically related to the epigenetic memory conferred by PcG repressors (Table 1). To explore this possibility, we examined whether *Pc* mutation could also enhance Hox induction by *dpp*>Trx in a similar manner. Compared to levels in the WT background, much more Scr, Ubx and Antp signals were induced by *dpp*>Trx in *Pc*^*4*^ mutant discs (S12A Fig). Moreover, the overall patterns of Hox signals were similar to those observed in *Hira* mutants. Consistently, severe phenotypes such as arista-to-leg and ESC were also seen (S12B Fig). We then monitored the sustainability of Hox expression in *Pc*^*4*^ mutants in pulse-chase experiments. Ubx signals remained detectable after a 60-h chase in *Pc*^*4*^ mutants, while Scr and Antp signals could last for at least 72 h in L2 and eye-antenna discs, respectively (Fig 6A). Similar to *Hira* mutants, a significant portion of Hox signals were located outside the region where *dpp*>GFP was normally seen. These results indicate that Hox genes induced from earlier stages can also be sustained in *Pc*^*4*^ mutants.

We further asked whether the genetic interaction between *Hira* and *Psc*—a member of the PcG repressors—could be manifested at larval stages. As expected, introduction of a *Psc* mutation resulted in more Ubx, Scr and Antp signals than *dpp*>Trx alone. Importantly, a combination of both *Psc* and *Hira* mutations resulted in even more Ubx, Scr and Antp signals in broader areas and higher RNA levels (Fig 6B and 6C), supporting a common role in Hox repression.

### H3.3 is required for restoration of the inactive state

**Fig 7:**
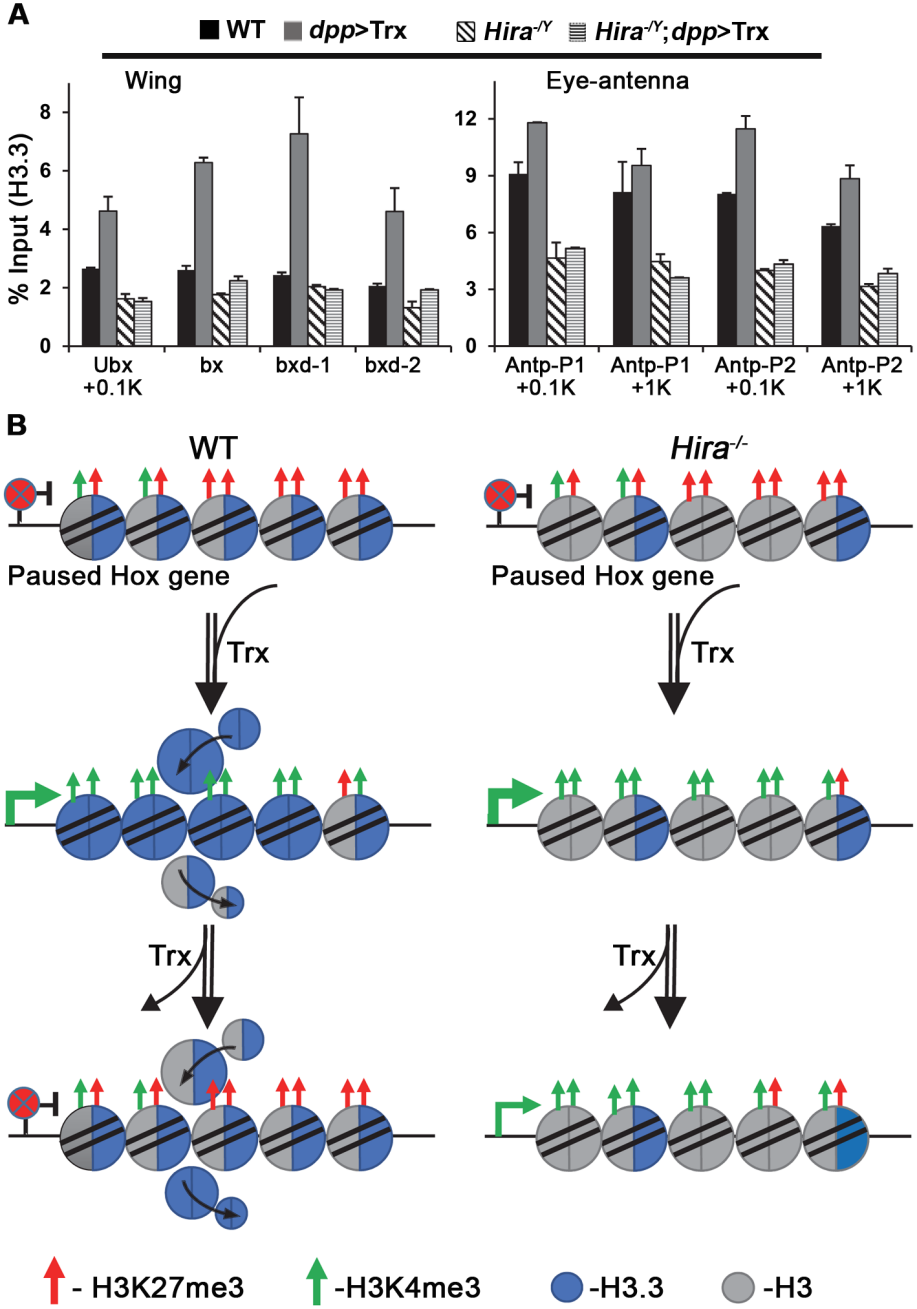
The involvement of H3.3 in maintenance of active Hox genes. (A) Reduced H3.3 on Hox genes of *Hira* mutants. ChIP assays were performed using anti-flag antibody for wing (left) and eye-antenna (right) discs expressing H3.3-Flag in different genotypes. In addition to promoter-proximal regions of *Ubx* and *Antp* (Figs 1E and 2F), the levels of H3.3 in three *Ubx* regulatory elements (bx, bxd-1, bxd-2) were measured (S2 Fig). Bar graphs represent the average ± SD of two separate ChIP experiments with two independent qPCR reactions. (B) A model for the dynamic conversion between paused and active states of Hox genes. Hox promoters are poised in the paused state in imaginal discs with flexible developmental potential. Relatively high levels of H3K27me3 over H3K4me3 is maintained in these genes. Introduction of extra doses of Trx by *dpp*>Trx shifts the equilibrium towards relatively higher levels of H3K4me3, resulting in conversion of the paused state to the active state. Concomitantly, the level of H3.3 is increased. The high level of H3.3 facilitates the replacement of active H3K4me3 marks after Trx withdrawal. Consequently, the active Hox promoters are rapidly reverted to the paused state. With less H3.3 or HIRA, the replacement of active H3K4me3 marks becomes less efficient. Accordingly, the active state of Hox genes can be maintained for longer periods. The relative abundances of H3K27me3 and H3K4me3 are indicated by the numbers of red and green arrows, respectively. Nucleosomes containing H3.3 or H3 are indicated by blue or grey circles, respectively. Hybrid nucleosomes containing both H3.3 and H3 are also indicated.

As a H3.3 chaperone, the main effect of HIRA on chromatin should be reflected by modulation of the level of H3.3. Using a constitutively active *Hsp83-H3*.*3 (Flag)* transgene introduced into different genetic backgrounds, we measured the levels of H3.3 on *Ubx* and *Antp* in wing and eye-antenna discs, respectively (Fig 7A). In wing discs, we monitored four regions, including the promoter-proximal region (+0.1 kb), an intragenic regulatory region at ~+25 kb (bx) [50, 51], and two upstream regulatory regions (bxd-1, bxd-2) at ~-28 kb and ~-29 kb [52, 53]. In the WT background, the level of H3.3 was substantially increased by *dpp*>Trx in all regions. As expected, relatively lower levels of H3.3 were detected in the promoter-proximal region of *Hira* mutants. However, no significant increase of H3.3 was induced by *dpp*>Trx in mutant discs, despite higher levels of RNA-Pol II and *Ubx* expression (Fig 2C and 2E). Compared to wing discs, relatively minor increases of H3.3 were induced by *dpp*>Trx in the +0.1 kb and +1 kb regions of the two *Antp* promoters in WT eye-antenna discs where no ectopic Antp signals were found. Similar to the *Ubx* promoter, the low levels of H3.3 detected in *Antp* promoters were not affected by *dpp*>Trx in mutant discs. Again, this was in sharp contrast to the induction of stronger Antp signals (Fig 2D). Although our results show that both H3.3 and HIRA are negatively involved in Hox gene regulation, the lack of a simple relationship between the level of H3.3 and Hox expression is inconsistent with a direct role in repression. Alternatively, H3.3-enriched regions have been recently reported to undergo faster turnover of chromatin marks [54, 55]. Therefore, our observations suggest that the low level of H3.3 in *Hira* mutants may defer the dynamic switch from active chromatin state to repressed state (see Discussion).

## Discussion

In this study, we show that the developmental restriction imposed on pluripotent imaginal tissues can be partially lifted through conversion of pre-existing transcriptional paused state of Hox genes into active elongation by simply increasing the level of a H3K4 methyltransferase. Furthermore, these developmental and molecular switches are regulated in tissue-specific manners by dynamic modulation of chromatin.

Mounting evidence suggests that the developmental potential of pluripotent progenitor cells is largely restricted by developmental regulators that are poised at a transcriptionally inactive paused state [1, 3, 4]. Our results support this view and extend it further with additional insights. We found that *Ubx*, *Scr* and *Antp* are poised at different transcriptional states in pluripotent imaginal discs. For example, *Ubx* possesses abundant RNA-Pol II Ser5P in the promoter-proximal region in wing discs, but little, if any, in L2 discs or eye-antenna discs. Although all these discs are formally considered inactive for *Ubx* expression, only wing discs exhibit features characteristic of the paused state. Similarly, *Scr* is poised at the paused state in L2/L3 discs, but not in eye-antenna or wing discs (S3B Fig). In contrast, *Antp* is kept at the paused state in eye-antenna discs. Thus, other than the paused state, an additional inactive state may exist. We refer to it as the “void state” for the lack of paused RNA-Pol II. Apparently, inactive Hox genes are in either paused or void states.

The paused state, rather than the void state, of Hox genes appears to correlate with the tendency of a given disc to adopt alternative developmental paths in animals with ectopic Hox expression [41, 42]. This relationship is further strengthened by our finding that *dpp*>Trx can induce similar developmental changes by converting the paused state of a specific Hox gene into the active state. In all cases examined, however, the void state remains refractory. Therefore, we suggest that developmental restriction can be effected by two mechanisms distinguishable by the presence or absence of paused RNA-Pol II of Hox genes. A given disc may adopt an alternative developmental path when the paused state of relevant Hox genes can be converted into the active state. In contrast, disc development becomes more restricted when Hox genes are in the void state.

It is generally believed that the paused state of Hox genes is stably maintained by PcG repressors through high levels of H3K27me3, a repressive histone mark [12, 56]. Our results clearly show that, for many discs, this state can be readily destabilized by an extra dose of Trx that gives rise to increases of active methylation marks such as H3K4me3. Concomitantly, the level of H3K27me3 is reduced. Although the direct cause of H3K27me3 reduction is unclear, the combined effects apparently results in greater increases of the relative ratio between active marks and repressive mark. Consequently, the paused state is converted into the active state. Conceptually, the paused state can be stably maintained by a constant ratio between functionally antagonistic marks. In support of this view, our results show that *dpp*>Trx fails to convert the paused state of *Antp* in eye-antenna discs in the absence of significant changes to these marks.

In addition to canonical histones, our results further show that non-canonical histones play important roles in maintaining the paused state of Hox genes, most evidently in eye-antenna discs. This role was initially revealed by the observation that mutations of two histone variants, H2A.Z and H3.3, and corresponding chaperones can cause substantial enhancement of various phenotypes mediated by ectopic Hox expression. Detailed analyses of *Hira* that encodes a H3.3 chaperone provided strong support to suggest that the enhanced Hox expression largely results from a prolonged active state via compromised nucleosome dynamics in *Hira* mutants. First, the extra Hox signals induced in the *Hira* background do not coincide with GFP signals induced by *dpp*>Gal4 (Fig 3A). Since GFP has a short half-life, its signals should match closely with the current activity of *dpp*>Gal4. Since the half-life of Trx is also short (~6 h), these deviant Hox signals, particularly for Antp, cannot be attributed to residual Gal4 or Trx in GFP-negative cells. Second, lineage tracing experiments revealed that cells showing deviant Hox signals may arise from those with active *dpp*>Gal4 as early as 48 h before the appearance of GFP-positive cells during the third instar. Since Hox signals can only last for about 12 h, these signals do not correspond to signals induced at earlier stages. Instead, they should result from continuous gene expression. Third, pulse-chase experiments showed that Hox genes, more extensively for *Scr* and *Antp*, are expressed for longer periods in mutant discs. For example, strong Antp signals were induced in mutant eye-antenna discs and remained obvious after a 48-h chase, whereas no signal was detected in WT discs (Fig 4C). Furthermore, we showed that comparable amounts of signals were induced in WT and mutant discs immediately after short pulses of *dpp*>Gal4 (Fig 5B). These results enabled us to deduce that the active state of Hox genes induced temporally by *dpp*>Gal4 can be maintained for longer periods in *Hira* mutants and that continuous Hox expression results in stronger cumulative effects.

Furthermore, our studies provide evidence to support a mechanistic link between the maintenance of the active state and the epigenetic memory conferred by trxG and PcG regulators [11, 57]. First, Hox expression was enhanced and maintained for longer periods in *Pc*^*4*^ mutants in a manner similar to that observed in *Hira* mutants. Second, strong genetic interactions were observed between *Hira* and *PcG* mutants (*Pc*^*4*^ and *Psc*^*1*^). Third, accompanied by Antp induction in mutant eye-antenna discs, the relative levels of H3K4me3 and H3K27me3 showed dynamic modulation. In light of the recent finding of physical association between HIRA and PcG [27], HIRA may be recruited by PcG. Previous studies have shown that H3.3 is enriched in chromatin regions that undergo rapid turnover to facilitate remodeling of chromatin landscapes [25, 58, 59]. Thus, the reduced levels of H3.3 observed in *Hira* mutants may slow down the turnover of pre-existing histone marks. Accordingly, H3K4me3 marks induced by *dpp*>Trx can be maintained much longer to allow continuous Hox expression.

For the conversion of the paused state to the active state, we propose a simple model for different situations (Fig 7B). At first, a Trx pulse can induce sufficient histone modification to destabilize the pre-existing balance between H3K4me3 and H3K27me3, and consequently promote active elongation of Hox genes through incorporation of more H3.3. In the absence of constant supply of Trx, the rapid turnover of active marks mediated by H3.3 results in reversion of the active state to the inactive paused state. However, with reduced levels of H3.3 or HIRA chaperone, the turnover of active marks becomes less efficient. Therefore, the active state can be maintained even after *dpp*>Trx is no longer expressed in those cells. We note that the unique property of each disc may render different responses to various regulatory factors. In addition, the presence of multiple cell lineages may result in variable responses in different parts of the same disc [6]. These complexities must be taken into consideration when cell-based models are being compared.

Recent findings that Trx is more active in catalyzing H3K4 mono-methylation than di- or tri-methylation suggest that our observations of increased H3K4me3 might not result solely from Trx activities [60–62]. It is possible that dSet1, the major *Drosophila* H3K4 trimethyltransferase [60, 61], is recruited to Hox genes and contributes significantly to the H3K4me3 increase. In addition, the ability of Trx to promote acetylation of H3K27 might also help antagonize the pre-existing balance between different methylation marks [13, 62]. It will be interesting to test these possibilities directly.

## Materials and Methods

### Fly stocks

*P(GSV6)GS12194/TM3* (*UAS-Trx*) was obtained from DGRC, Kyoto. *yw;dom*^*3*^*/SM6a*, *yw;dom*^*9*^*/CyO*, *yw;Spt5*^*MGE-3*^*/SM1*, *w;nej*^*P*^*/FM7c*, *w;ash1*^*22*^*/TM6C*, *Psc*^*1*^/*CyO*, *Pc*^*4*^*/TM3*, *w;His2Av*^*810*^*/TM3*, *P{Gal4}A9*, *w;dpp*^*blk*^*-Gal4/TM3* [40], *en-Gal4/CyO*, *act-Gal4/TM3*, *UAS-Antp/TM3*, *UAS-Scr/CyO*, and *tub-Gal80*^*ts*^*/CyO* were obtained from BDSC, Indiana. RNAi lines (listed in S1 Table) were obtained from VDRC, Vienna. *Hira*^*ssm*^*/FM7i* [29], *Hira*^*HR1*^*/FM7c* [33], *ywHis3*.*3B*^*0*^, *His3*.*3A*^*2X1*^*/CyO* [34], *Hsp83-H3*.*3(Flag)* [63] and G-TRACE lines consisting of *UAS-RedStinger* (RFP) *UAS-FLP Ubi-p63E(FRT*.*STOP)-Stinger* (GFP)*/CyO* [49] were obtained from individual labs as indicated.

### Genetic crosses

Flies were raised at 25°C on standard food unless otherwise mentioned. Homozygous *UAS-Trx* virgin females were crossed to *dpp-Gal4/TM6B*,*Tb* males at 25°C unless otherwise specified. Adult phenotypes of *dpp-Gal4/UAS-Trx* were scored three days after eclosion. In pulse-chase experiments, virgin females carrying *UAS* transgenes with or without mutations were crossed to *tub-Gal80*^*ts*^;*dpp-Gal4* males and kept at either 29°C or 21°C for indicated periods to inactivate or activate Gal80^ts^, respectively. Flies were transferred every 12 h. The phenotypes wing-to-haltere, arista-to-antenna and sex comb number on L2 and L3 were assessed three days after flies hatched. For genetic interactions, virgin females heterozygous for *Hira*^*ssm*^, *Hira*^*HR1*^, *H2Av*^*810*^, *dom*^*9*^, *dom*^*3*^, *His3*.*3A*^*2X1*^, *His3*.*3B*^*0*^ mutants were crossed to *Pc*^*4*^ heterozygous males. The number of sex comb teeth on L2 and L3 of their male progeny were counted as described previously [64]. The statistical analyses were performed by R language (http://www.r-project.org/). One-sided Wilcoxon rank sum test was used to compute the statistical significance for the increased sex comb number.

### Immunostaining of discs

Imaginal discs isolated from third instar larvae carrying different genotypes were fixed by 3.7% formaldehyde in PBS for 20 min. Discs were washed three times with PBT (0.15% Triton X, 0.2% NP40, 0.2% Tween 20 in PBS) for 5 min. After blocking in a PBT solution containing 3% bovine serum albumin (Sigma-Aldrich) and 5% goat normal serum (Vector Laboratories) for 1 h, discs were incubated overnight at 4°C with primary antibodies. Following six washes with PBT for 5 min, discs were incubated in the dark with fluorescently-labelled secondary antibodies for 1 h at room temperature and washed three times for 5 min with PBT. Samples were then stained with Hoechst 33258 (0.5 μg/mL) (Molecular Probe) for 15 min, followed by six washes for 5 min with PBT. Before mounting in Vectashield (Vector Laboratories), samples were soaked in 50% glycerol in PBS for 30 min. As for primary antibodies, culture supernatants were used. Anti-Ubx (FP3.38; 1:2) and anti-Scr (6H4B; 1:2) supernatants were prepared in our lab [64], whereas anti-Antp (4C13; 1:4) was obtained from Developmental Studies Hybridoma Bank. The secondary antibodies were conjugated with Rhodamine Red-X and Cy5 for the anti-mouse and anti-rabbit antibodies, respectively (Jackson ImmunoResearch). All confocal images were taken using an LSM510Meta microscope (Zeiss) at 20X magnification. Images were aligned and processed using Adobe Photoshop CS3.

### Chromatin immunoprecipitation assays (ChIP)

ChIP of imaginal discs was performed as described previously [65] with some modifications. Details are available in the supplementary information (S1 Text). Specific antibodies for Ser5P (H14, Covance), Ser2P (H5, Covance), rabbit H3K4me3 (ab8580, Abcam), rabbit H3K27me3 (39156, Active motif), and anti-Flag (F-3165, M2, Sigma) were used for ChIP experiments. Quantitative PCR was performed in triplicate using SYBR Green at standard settings (ABI 7500, Applied Bioscience). Data are represented as percentage DNA present in input material. Bar graphs in Figs 1, 2 and 7 represent the averages ± SD values obtained from two independent experiments repeated twice.

### RT-qPCR

Total RNAs from 20–30 wing, second leg or eye-antenna imaginal discs were prepared from L3 of different genotypes by RNeasy protocol (QIAGEN). After reverse-transcription, cDNAs were measured by quantitative PCR according to the vendor’s protocol, using SYBR Green at standard settings (ABI 7500, Applied Bioscience). Two separate experiments with each in triplicate were performed. Primers are listed in S4 Table.

## Supporting Information

S1 Fig Hox induction in restricted regions of imaginal discs. (A) Restricted Ubx induction in wing discs. Four Gal4 lines (*actin*, *dpp*, *A9*, *engrailed*) were used to drive Trx and GFP simultaneously. Their effects on GFP and *Ubx* expression in discs and adult wings are shown. GFP (green) and Ubx signals (red) are shown in separate panels or together with DNA staining (blue). Adult wings resulting from *dpp-*, *A9-*, and *engrailed*-driven Trx are shown. *actin*-driven Trx causes pupal lethality. A schematic representation of anterior/posterior (A/P) and dorsal/ventral (D/V) compartments and their boundaries is shown. White dots outline the pouch region. Scale bars in this and subsequent confocal images represents 50 μm. (B) Restricted Scr induction in leg discs. The patterns of Scr induction in L1, L2 and L3 leg discs from *actin*>GFP (left panel) or *actin*>Trx, GFP (right panel) larvae are compared. Ectopic Scr signals are seen in regions forming adult tarsal segments. Individual or merged images of Scr (red), GFP (green) and DNA (blue) are shown. Anterior/Posterior (A/P) compartments are indicated.
(TIF)

S2 Fig Gene organization of *Scr*, *Ubx* and *Antp*. Both *Scr* and *Antp* contain two separate promoters and start sites. The direction of transcription is indicated by rightward arrows. The non-coding and coding exons are indicated by grey and orange boxes, respectively. Introns are indicated by thin lines. Approximate locations of primers used in this study are indicated by red (ChIP) or blue bars (RT-qPCR) below the gene map. Magnified views of the TSS regions of *Ubx* and *Antp* are shown. P1 and P2 correspond to the proximal regions of promoters 1 and 2, respectively. +0.1K and +1K correspond to regions about 100 bp and 1 kb downstream of the TSS, respectively. 3’ corresponds to the gene terminus. bxd-1, bxd-2 and bx correspond to *Ubx* distal regulatory elements. P1, P2 and 3’ end primers were used to measure RNA-Pol II occupancy, whereas other primers were used to measure tri-methylated H3 or H3.3. Primer sequences are listed in S4 Table. The upstream-most start sites are used as the reference point for the scales shown above genes.
(PDF)

S3 Fig Hox genes in the void state. (A) Analyses of Ser5P enrichment in *Scr* or *Ubx* in different leg discs. ChIP analyses of promoter and 3’ regions of *Scr* or *Ubx* were performed with a Ser5P antibody for samples prepared from WT L2 (left) or L3 discs (right). (B) Analyses of Ser5P and Ser2P enrichment of *Scr* or *Ubx* in wing or eye-antenna discs. Chromatin samples prepared from wing (left) or eye-antenna discs (right) of the following genetic backgrounds were subjected to ChIP analyses with antibodies against Ser5P or Ser2P: WT, *dpp*>Trx, *Hira*^*-/Y*^, *Hira*^*-/Y*^*;dpp*>Trx. The bar graphs represent the averages ± SD of two independent ChIP experiments, each with two separate qPCR reactions. The scale on the y-axis corresponds to 1% of input chromatin. Analyzed regions are described in S2 Fig.
(PDF)

S4 Fig Modifier screening for *dpp*>Trx phenotypes. Based on the wing phenotype induced by *dpp*>Trx, a modifier screening was conducted with co-expression of the RNAi lines listed in S1 Table. In no case was the small wing induced by *dpp*>Trx further reduced. The effects of RNAi lines were evaluated by the degree of recovery to full wing size as indicated by the number of “+” signs. The “-” sign indicates no effect. Scale bar, 0.3 mm.
(PDF)

S5 Fig Enhanced ESC phenotype by histone chaperone or variant mutations. Transformation of adult L2/3 to L1 was scored in a heterozygous *Pc* background with other mutants. Each bar represents the percentage of ESCT transformation with respect to the number of ESCT (x-axes). Note that the percentage transformation increased with increased ESCT when *Pc* and other mutants were combined. *p-* and W-value were calculated based on one-sided Wilcoxon rank sum test.
(TIF)

S6 Fig Enhanced *Scr* phenotypes in *Hira* and *dom* mutants. (A) Adult leg phenotype. WT, *Hira* and *dom* adults mutants exhibit sex combs only on the first leg (L1, arrow). (B) Extra sex combs on adult legs. The sex combs induced in adult L2 (upper) and L3 (lower) by *dpp*>Trx are enhanced in *Hira* and *dom* mutants. The average number of sex comb teeth is shown and the numbers of legs scored are in parentheses. (C) Ectopic Scr expression in leg discs. Upper panel, Scr expression induced in L2 discs by *dpp*>Trx in WT, *Hira* or *dom* mutant backgrounds. Lower panel, merged images for Scr (red), GFP (green) and DNA (blue) as described previously. (B, C) Experiments were carried out at 21°C. (D) Bar graph depicts phenotypic variation as described previously.
(TIF)

S7 Fig Lineage tracing for *dpp*-Gal4 activity during development. (A) Schematic diagram of components employed in the G-TRACE strategy [49]. *UAS-RFP* and *UAS-FLP* are directly under the control of *dpp*-Gal4, which is active primarily in larval discs. Nuclear EGFP (*nEGFP*) is linked to the *Ubi-p63E* promoter with a cassette containing a transcriptional termination signal flanked by *FRT*. Through the removal of *FRT* by FLP recombinase, rearranged *Ubi-p63E-nEGFP* is constitutively activated, thus marking all descendent cells. (B) Lineage tracing of *dpp*-Gal4 in imaginal discs. Experiments were performed in the presence of *tub-Gal80*^*ts*^ as described in Fig 3C. Gal80^ts^ is inactivated for different lengths at 29°C and then re-activated by shifting to 21°C for indicated hours. *dpp*-Gal4 becomes activated reciprocally. Due to its rapid degradation, strong RFP signals can be seen only in cells that remain active for *dpp*-Gal4. In contrast, all cells that have ever been active for *dpp*-Gal4 are marked by GFP signals. Hours refer to the duration of chase described in Fig 3C. Individual RFP (red), GFP (green) or a merge panel with DNA (blue) are shown for leg (left), wing (middle) and eye-antenna discs (right). Arrowheads indicate regions with deviant Hox expression in Fig 3A. Note that most GFP signals appear during the 48–72 h interval.
(TIF)

S8 Fig Enhanced ESC phenotype by *dpp*>Trx pulse. Bar graphs are as described previously. Hours indicate the duration of Trx pulse (see Fig 3C). At any given time points, the sex combs induced in adult L2 and L3 by *dpp*>Trx are enhanced in *Hira* mutants.
(TIF)

S9 Fig Enhanced ESC phenotype by *dpp*>Trx short pulse. Bar graph is as described previously. Hours indicate the duration of Trx pulses (see Fig 5A). For both 24 and 48 h, the sex combs induced in adult L2 and L3 by *dpp*>Trx are enhanced in *Hira* mutants.
(TIF)

S10 Fig Stability of induced Hox proteins. (A) Stability of ectopic Scr in L2 discs. Pulse-chase experiments were performed for animals carrying *tub-Gal80*^*ts*^; *dpp*-*Gal4*, *UAS-Scr in* WT (left) or *Hira* backgrounds (right) with different lengths of chase as indicated in Fig 3C. Scr staining in L2 discs is shown alone or together with DNA. Note that similar results were observed in WT or *Hira* mutants. (B) Stability of ectopic Antp in eye-antenna discs. Similar pulse-chase experiments were performed for animals carrying *tub-Gal80*^*ts*^; *dpp*-*Gal4*, *UAS*-*Antp*. Antp staining in eye-antenna discs is shown alone or together with DNA (blue). In all cases, ectopic Scr and Antp signals disappeared within 12 h of chase.
(TIF)

S11 Fig Stability of induced Trx. Experiment was carried out as in Fig 3C. Pulse-chase experiments were performed for animals carrying *tub-Gal80*^*ts*^; *dpp*-*Gal4*; *UAS*-*Trx*, *GFP*. Duration of the chase is indicated. Trx (red) and GFP (green) signals in wing, eye-antenna and L2 discs are shown alone or together with DNA (blue). Note that endogenous Trx expression is uniform in all discs and that induced Trx signals disappear faster than GFP.
(TIF)

S12 Fig Enhanced phenotypes of *dpp*>Trx in *Pc* mutants. (A) Ectopic Hox expression in *Pc*^*4*^ heterozygote mutants. Antp, Ubx and Scr expression patterns in *Pc*^*-/+*^ (left) or *Pc*^*-/+*^;*dpp*>Trx (right) backgrounds are shown for eye-antenna, wing and L2 disc, respectively. Arrowheads indicate regions with deviant Hox expression in Fig 3A. For comparison, Hox expression induced by *dpp*>Trx alone is shown (Figs 2C, 2D and S6C). (B) Enhanced adult phenotypes in *Pc*^*4*^ heterozygote mutants. Arista-to-leg transformation is seen in all *Pc*^*-/+*^;*dpp*>Trx adults (left panel). The percentages of transformed *dpp*>Trx adults are indicated in parentheses. Strong transformation of L2/L3 is seen in *Pc*^*-/+*^;*dpp*>Trx adults (right panel). The average numbers of sex comb teeth in *Pc*^*-/+*^;*dpp*>Trx are indicated for L2 and L3. The corresponding numbers in *Pc*^*-/+*^ mutants are indicated in parentheses. Scale bar, 0.1 mm.
(PDF)

S1 Table Wing phenotypes in RNAi backgrounds. (PDF)

S2 Table Genetic interaction between *Pc* and histone chaperone or variant mutations. (PDF)

S3 Table Induction of arista-to-leg transformation. (PDF)

S4 Table List of primers. (PDF)

S1 Text Chromatin immunoprecipitation assays (ChIP). (PDF)
