# Supplementary figures and images for: Maintenance of Tissue Pluripotency by Epigenetic Factors Acting at Multiple Levels
Source: PLoS Genet. 2016 Feb 29;12(2):e1005897. doi: 10.1371/journal.pgen.1005897 (PMC4771708; doi:10.1371/journal.pgen.1005897)

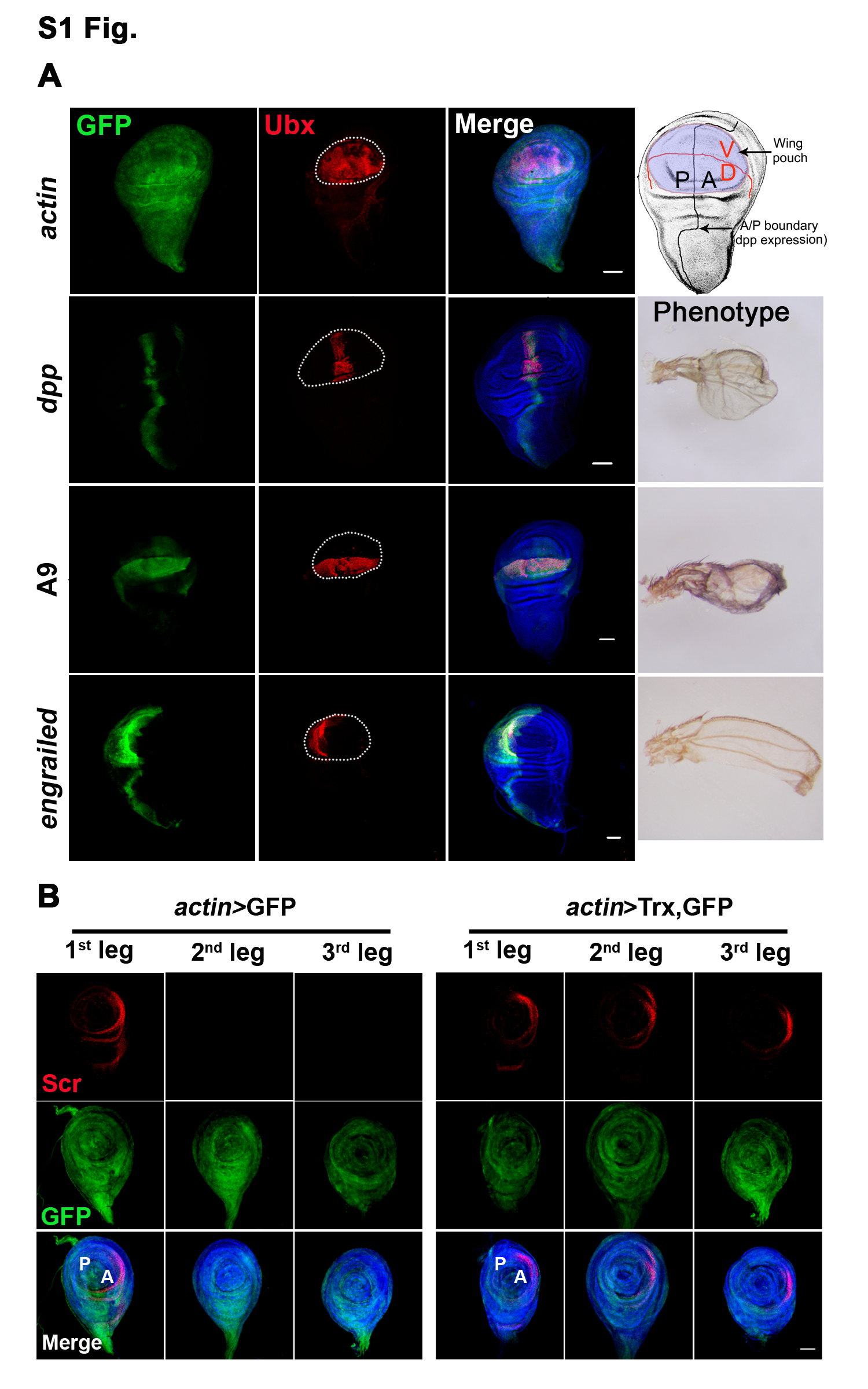

Supplement: S1 Fig — (A) Restricted Ubx induction in wing discs. Four Gal4 lines (actin, dpp, A9, engrailed) were used to drive Trx and GFP simultaneously. Their effects on GFP and Ubx expression in discs and adult wings are shown. GFP (green) and Ubx signals (red) are shown in separate panels or together with DNA staining (blue). Adult wings resulting from dpp-, A9-, and engrailed-driven Trx are shown. actin-driven Trx causes pupal lethality. A schematic representation of anterior/posterior (A/P) and dorsal/ventral (D/V) compartments and their boundaries is shown. White dots outline the pouch region. Scale bars in this and subsequent confocal images represents 50 μm. (B) Restricted Scr induction in leg discs. The patterns of Scr induction in L1, L2 and L3 leg discs from actin>GFP (left panel) or actin>Trx, GFP (right panel) larvae are compared. Ectopic Scr signals are seen in regions forming adult tarsal segments. Individual or merged images of Scr (red), GFP (green) and DNA (blue) are shown. Anterior/Posterior (A/P) compartments are indicated. (TIF) [file pgen.1005897.s001.tif]

S2 Fig.

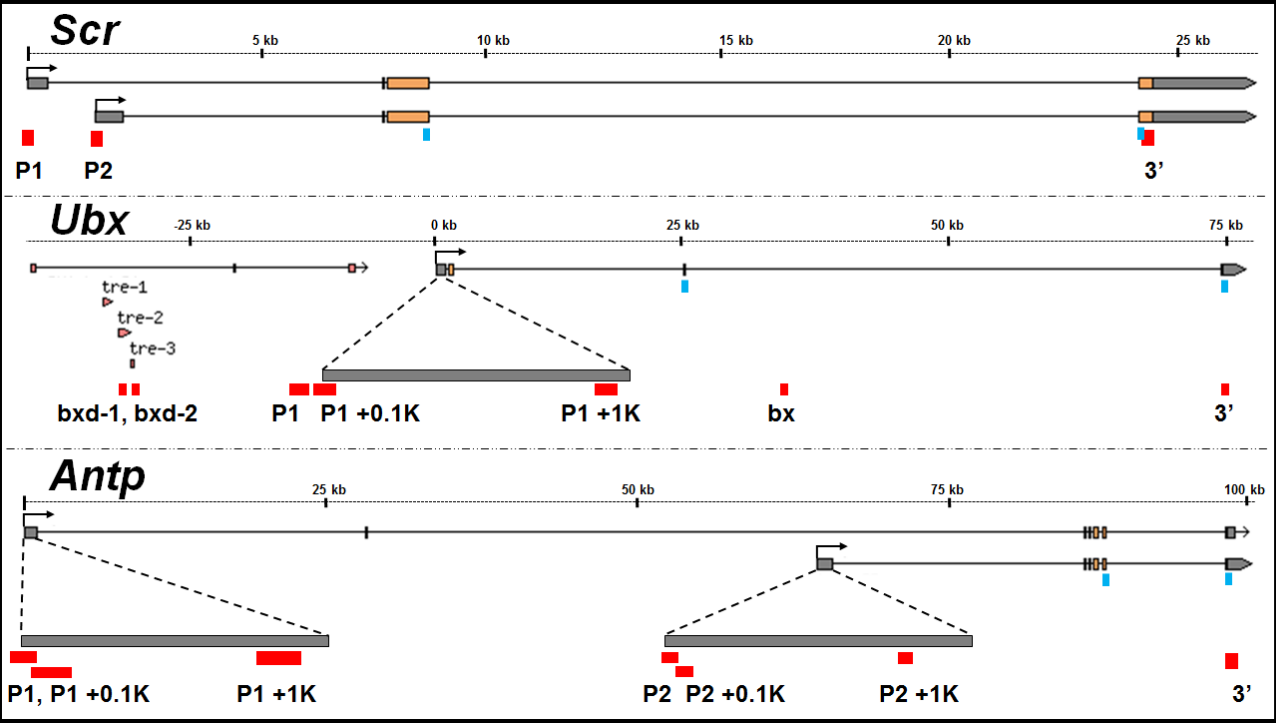

Supplement: S2 Fig — Both Scr and Antp contain two separate promoters and start sites. The direction of transcription is indicated by rightward arrows. The non-coding and coding exons are indicated by grey and orange boxes, respectively. Introns are indicated by thin lines. Approximate locations of primers used in this study are indicated by red (ChIP) or blue bars (RT-qPCR) below the gene map. Magnified views of the TSS regions of Ubx and Antp are shown. P1 and P2 correspond to the proximal regions of promoters 1 and 2, respectively. +0.1K and +1K correspond to regions about 100 bp and 1 kb downstream of the TSS, respectively. 3’ corresponds to the gene terminus. bxd-1, bxd-2 and bx correspond to Ubx distal regulatory elements. P1, P2 and 3’ end primers were used to measure RNA-Pol II occupancy, whereas other primers were used to measure tri-methylated H3 or H3.3. Primer sequences are listed in S4 Table. The upstream-most start sites are used as the reference point for the scales shown above genes. (PDF) [file pgen.1005897.s002.pdf]

**S3 Fig.**

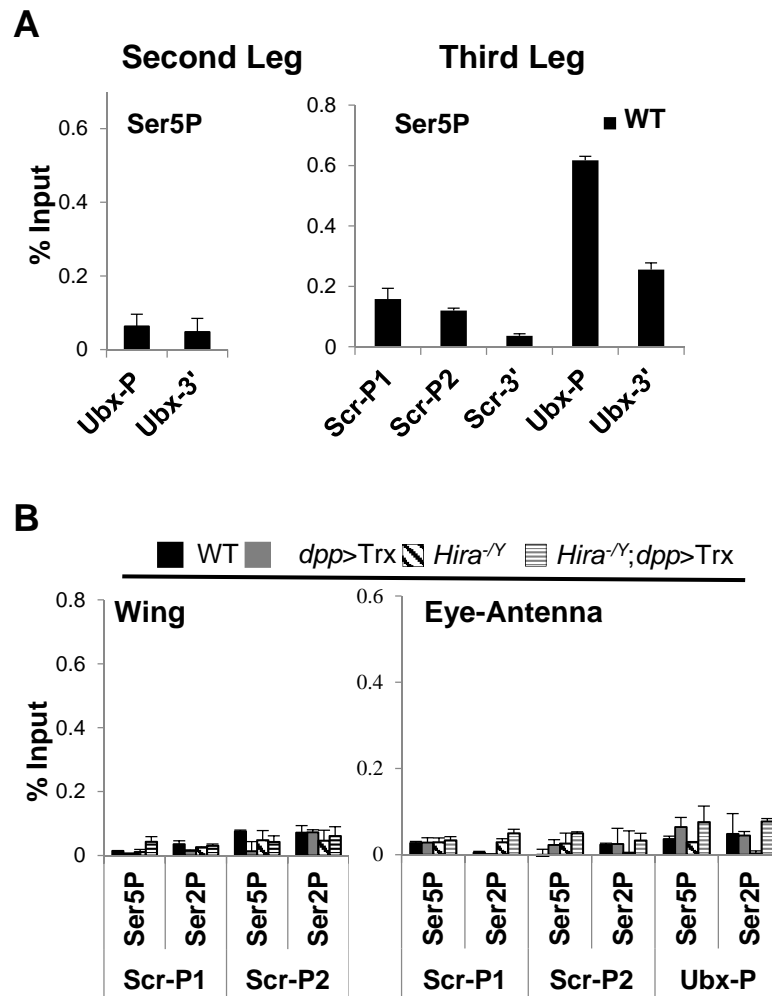

Supplement: S3 Fig — (A) Analyses of Ser5P enrichment in Scr or Ubx in different leg discs. ChIP analyses of promoter and 3’ regions of Scr or Ubx were performed with a Ser5P antibody for samples prepared from WT L2 (left) or L3 discs (right). (B) Analyses of Ser5P and Ser2P enrichment of Scr or Ubx in wing or eye-antenna discs. Chromatin samples prepared from wing (left) or eye-antenna discs (right) of the following genetic backgrounds were subjected to ChIP analyses with antibodies against Ser5P or Ser2P: WT, dpp>Trx, Hira-/Y, Hira-/Y;dpp>Trx. The bar graphs represent the averages ± SD of two independent ChIP experiments, each with two separate qPCR reactions. The scale on the y-axis corresponds to 1% of input chromatin. Analyzed regions are described in S2 Fig. (PDF) [file pgen.1005897.s003.pdf]

S4 Fig.

RNAi; *dpp*>Trx

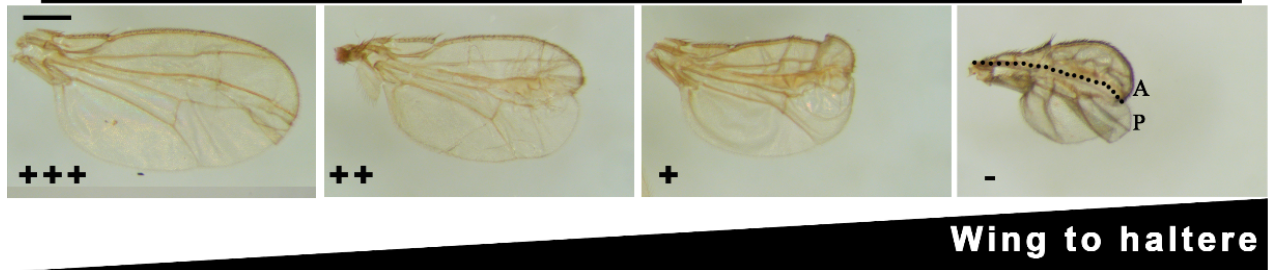

Supplement: S4 Fig — Based on the wing phenotype induced by dpp>Trx, a modifier screening was conducted with co-expression of the RNAi lines listed in S1 Table. In no case was the small wing induced by dpp>Trx further reduced. The effects of RNAi lines were evaluated by the degree of recovery to full wing size as indicated by the number of “+” signs. The “-” sign indicates no effect. Scale bar, 0.3 mm. (PDF) [file pgen.1005897.s004.pdf]

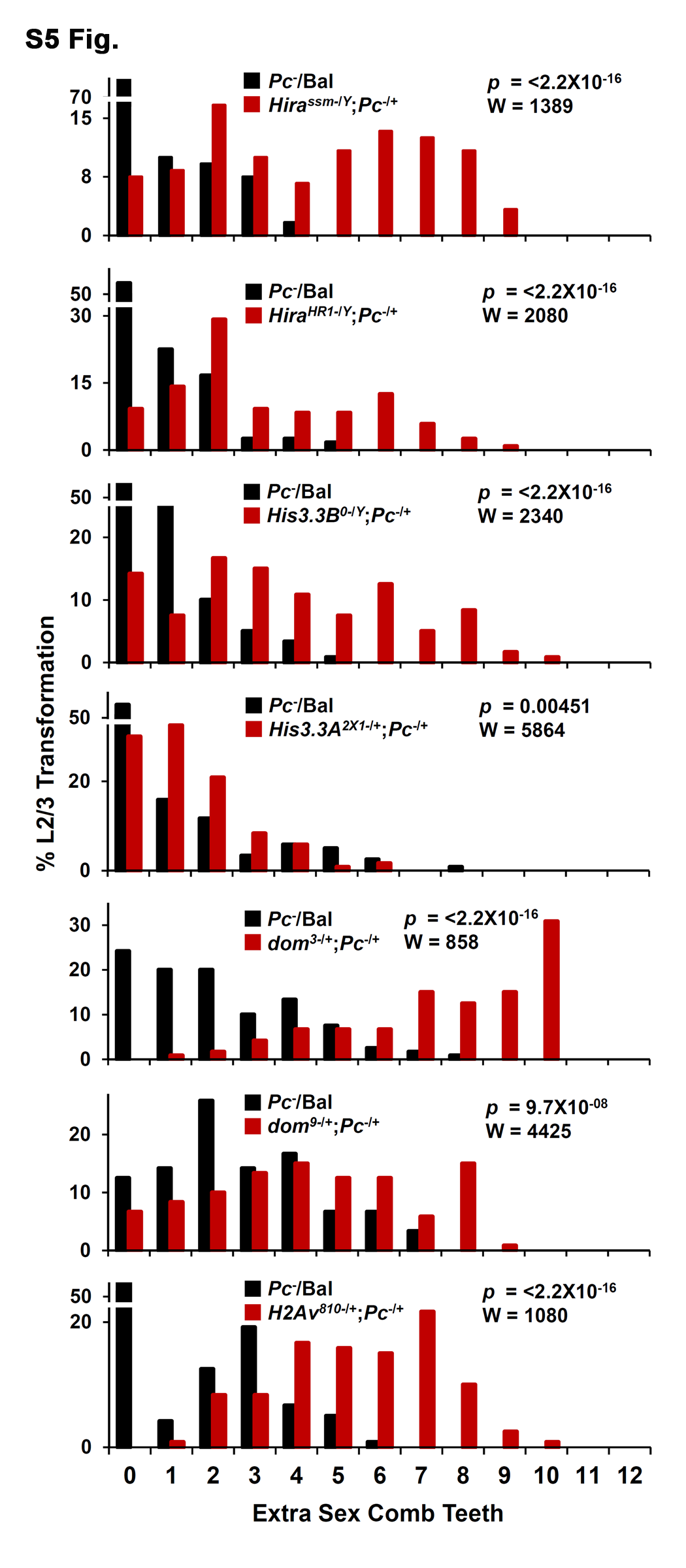

Supplement: S5 Fig — Transformation of adult L2/3 to L1 was scored in a heterozygous Pc background with other mutants. Each bar represents the percentage of ESCT transformation with respect to the number of ESCT (x-axes). Note that the percentage transformation increased with increased ESCT when Pc and other mutants were combined. p- and W-value were calculated based on one-sided Wilcoxon rank sum test. (TIF) [file pgen.1005897.s005.tif]

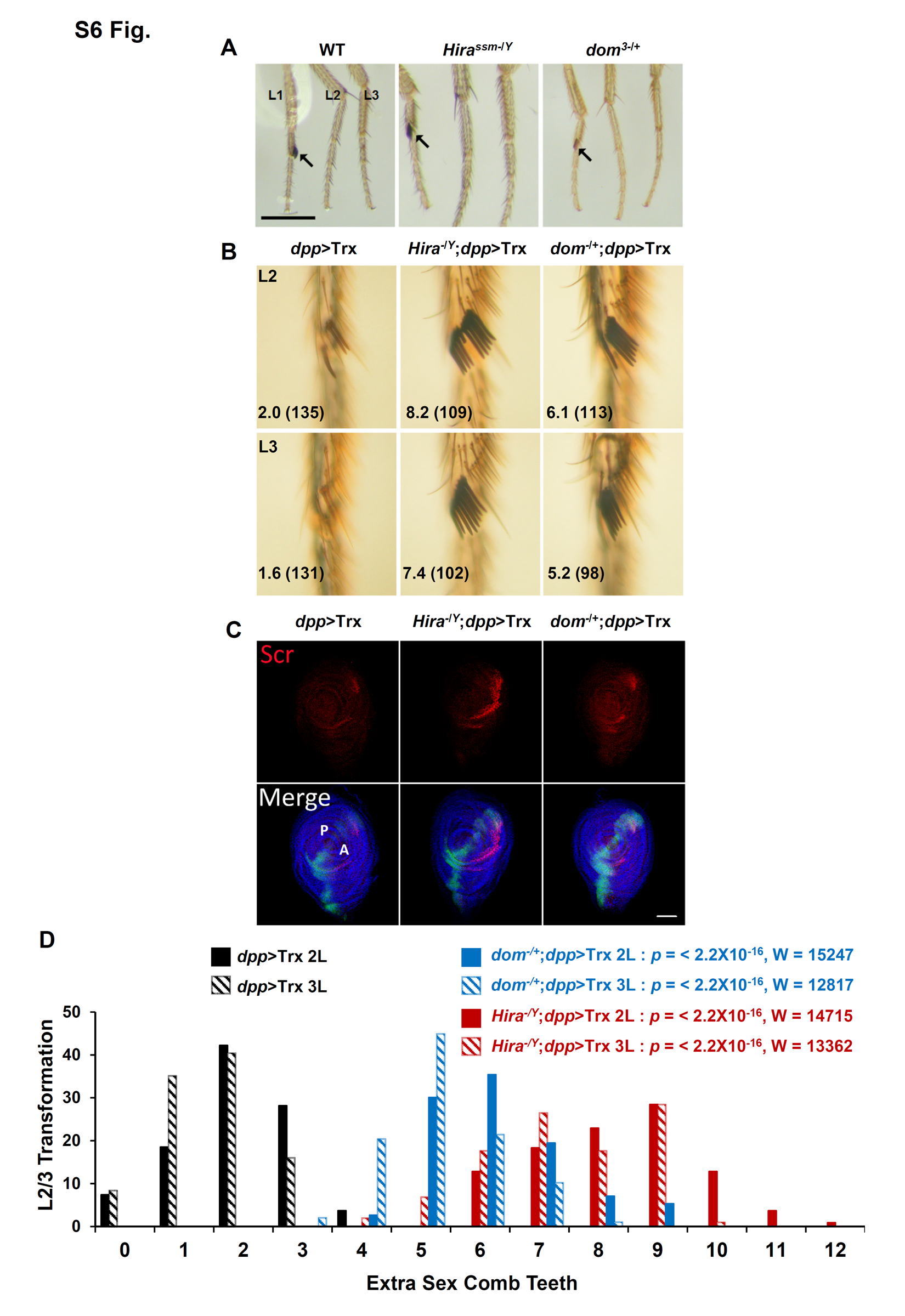

Supplement: S6 Fig — (A) Adult leg phenotype. WT, Hira and dom adults mutants exhibit sex combs only on the first leg (L1, arrow). (B) Extra sex combs on adult legs. The sex combs induced in adult L2 (upper) and L3 (lower) by dpp>Trx are enhanced in Hira and dom mutants. The average number of sex comb teeth is shown and the numbers of legs scored are in parentheses. (C) Ectopic Scr expression in leg discs. Upper panel, Scr expression induced in L2 discs by dpp>Trx in WT, Hira or dom mutant backgrounds. Lower panel, merged images for Scr (red), GFP (green) and DNA (blue) as described previously. (B, C) Experiments were carried out at 21°C. (D) Bar graph depicts phenotypic variation as described previously. (TIF) [file pgen.1005897.s006.tif]

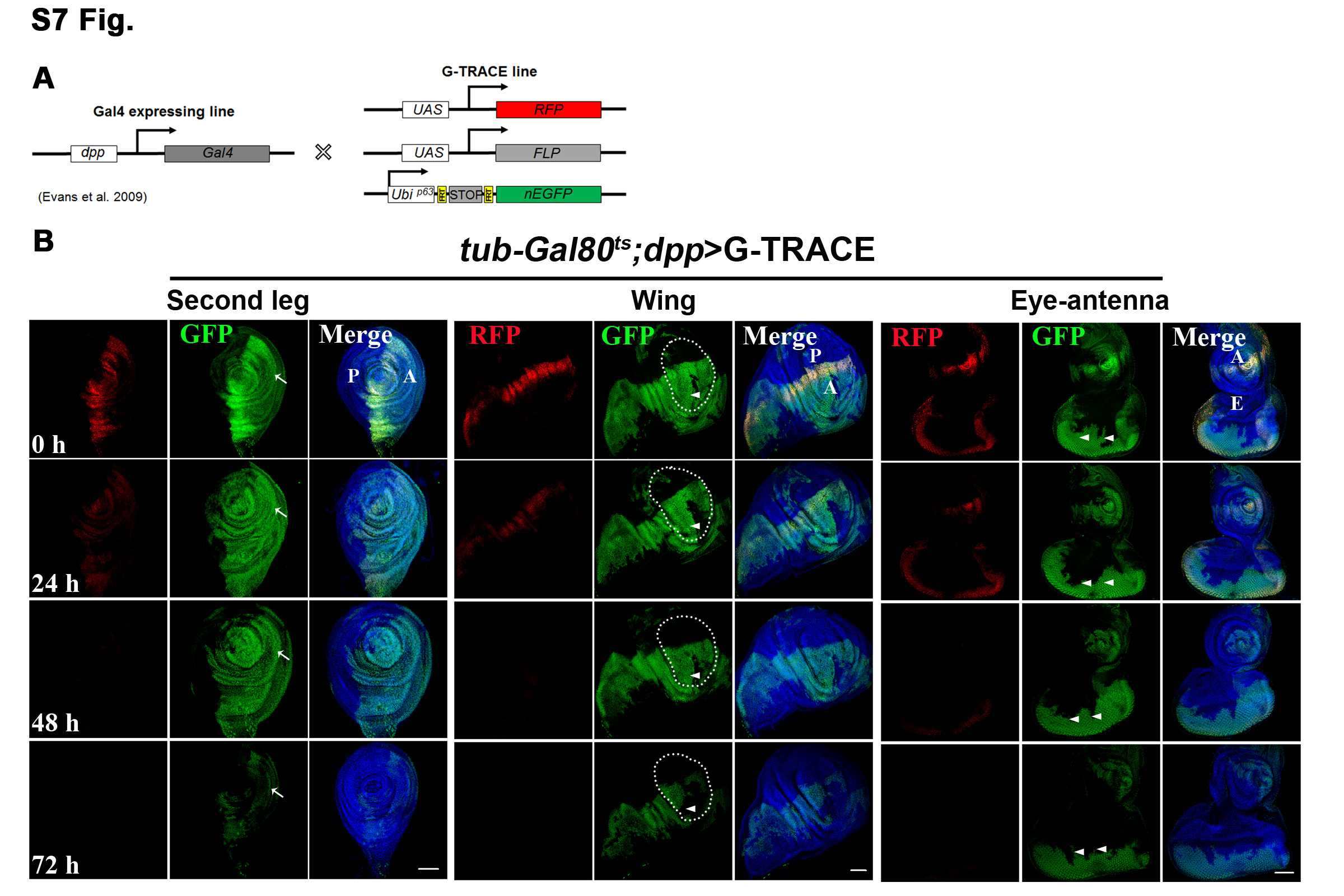

Supplement: S7 Fig — (A) Schematic diagram of components employed in the G-TRACE strategy [49]. UAS-RFP and UAS-FLP are directly under the control of dpp-Gal4, which is active primarily in larval discs. Nuclear EGFP (nEGFP) is linked to the Ubi-p63E promoter with a cassette containing a transcriptional termination signal flanked by FRT. Through the removal of FRT by FLP recombinase, rearranged Ubi-p63E-nEGFP is constitutively activated, thus marking all descendent cells. (B) Lineage tracing of dpp-Gal4 in imaginal discs. Experiments were performed in the presence of tub-Gal80ts as described in Fig 3C. Gal80ts is inactivated for different lengths at 29°C and then re-activated by shifting to 21°C for indicated hours. dpp-Gal4 becomes activated reciprocally. Due to its rapid degradation, strong RFP signals can be seen only in cells that remain active for dpp-Gal4. In contrast, all cells that have ever been active for dpp-Gal4 are marked by GFP signals. Hours refer to the duration of chase described in Fig 3C. Individual RFP (red), GFP (green) or a merge panel with DNA (blue) are shown for leg (left), wing (middle) and eye-antenna discs (right). Arrowheads indicate regions with deviant Hox expression in Fig 3A. Note that most GFP signals appear during the 48–72 h interval. (TIF) [file pgen.1005897.s007.tif]

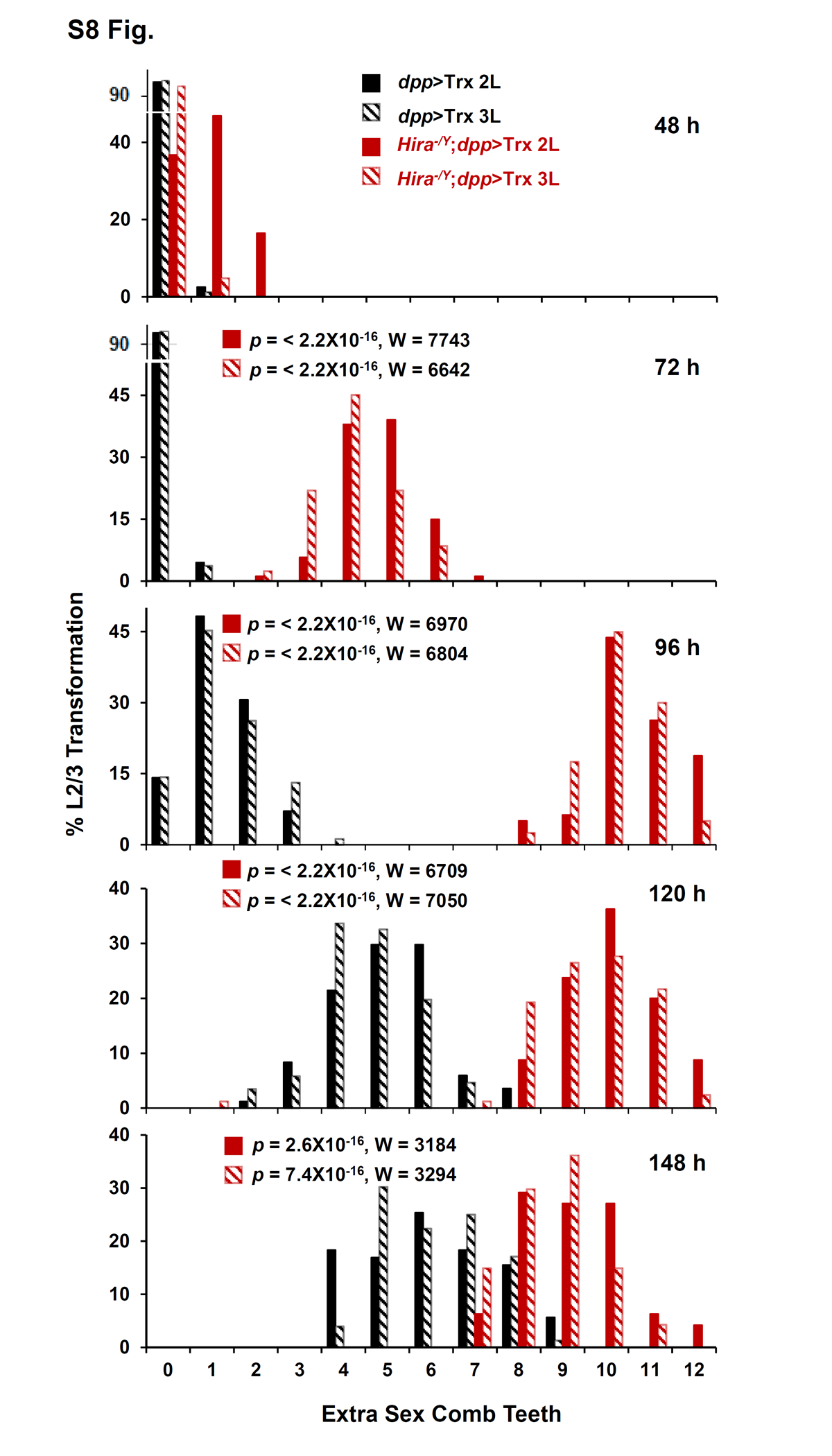

Supplement: S8 Fig — Bar graphs are as described previously. Hours indicate the duration of Trx pulse (see Fig 3C). At any given time points, the sex combs induced in adult L2 and L3 by dpp>Trx are enhanced in Hira mutants. (TIF) [file pgen.1005897.s008.tif]

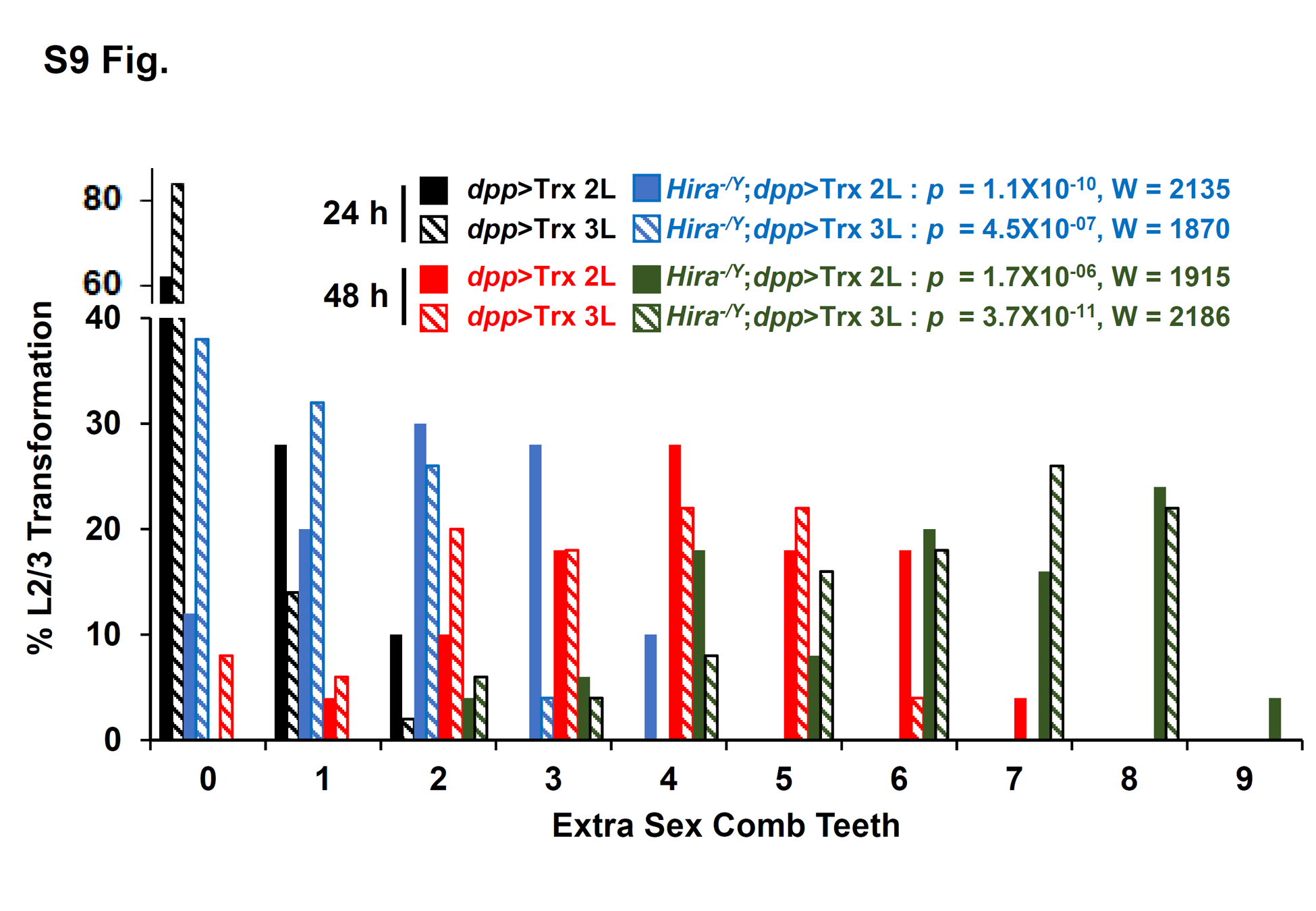

Supplement: S9 Fig — Bar graph is as described previously. Hours indicate the duration of Trx pulses (see Fig 5A). For both 24 and 48 h, the sex combs induced in adult L2 and L3 by dpp>Trx are enhanced in Hira mutants. (TIF) [file pgen.1005897.s009.tif]

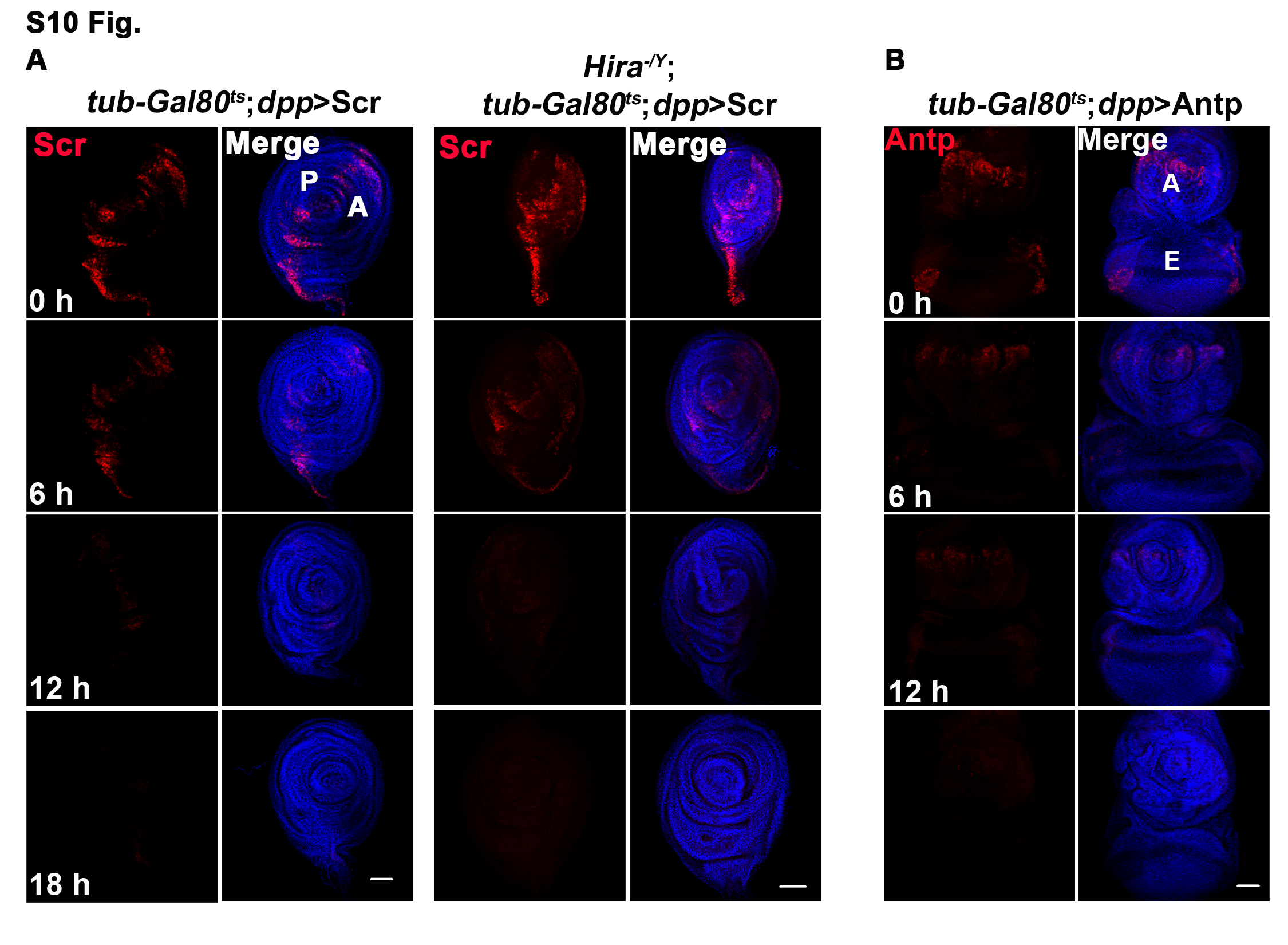

Supplement: S10 Fig — (A) Stability of ectopic Scr in L2 discs. Pulse-chase experiments were performed for animals carrying tub-Gal80ts; dpp-Gal4, UAS-Scr in WT (left) or Hira backgrounds (right) with different lengths of chase as indicated in Fig 3C. Scr staining in L2 discs is shown alone or together with DNA. Note that similar results were observed in WT or Hira mutants. (B) Stability of ectopic Antp in eye-antenna discs. Similar pulse-chase experiments were performed for animals carrying tub-Gal80ts; dpp-Gal4, UAS-Antp. Antp staining in eye-antenna discs is shown alone or together with DNA (blue). In all cases, ectopic Scr and Antp signals disappeared within 12 h of chase. (TIF) [file pgen.1005897.s010.tif]

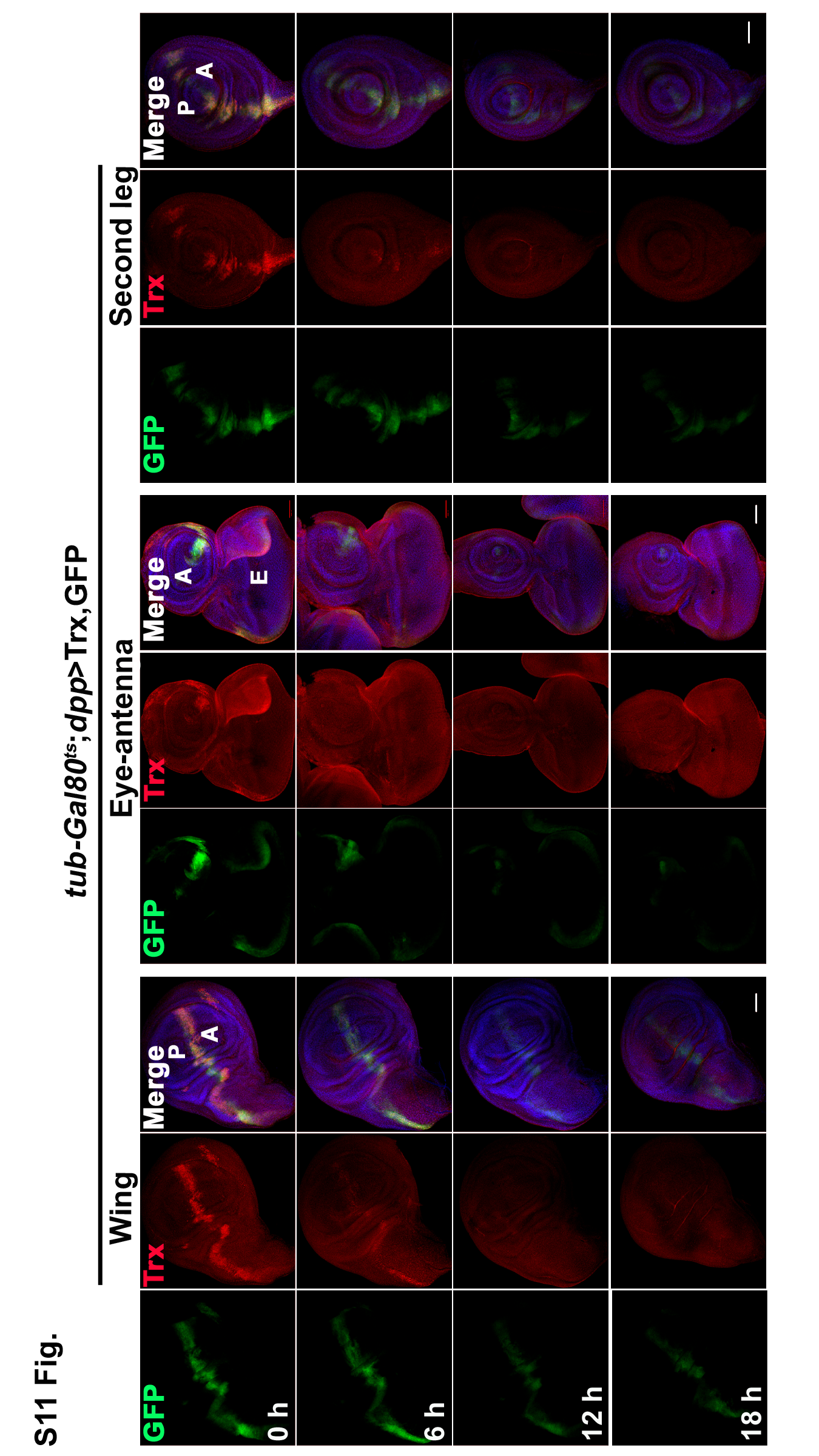

Supplement: S11 Fig — Experiment was carried out as in Fig 3C. Pulse-chase experiments were performed for animals carrying tub-Gal80ts; dpp-Gal4; UAS-Trx, GFP. Duration of the chase is indicated. Trx (red) and GFP (green) signals in wing, eye-antenna and L2 discs are shown alone or together with DNA (blue). Note that endogenous Trx expression is uniform in all discs and that induced Trx signals disappear faster than GFP. (TIF) [file pgen.1005897.s011.tif]

S12 Fig.

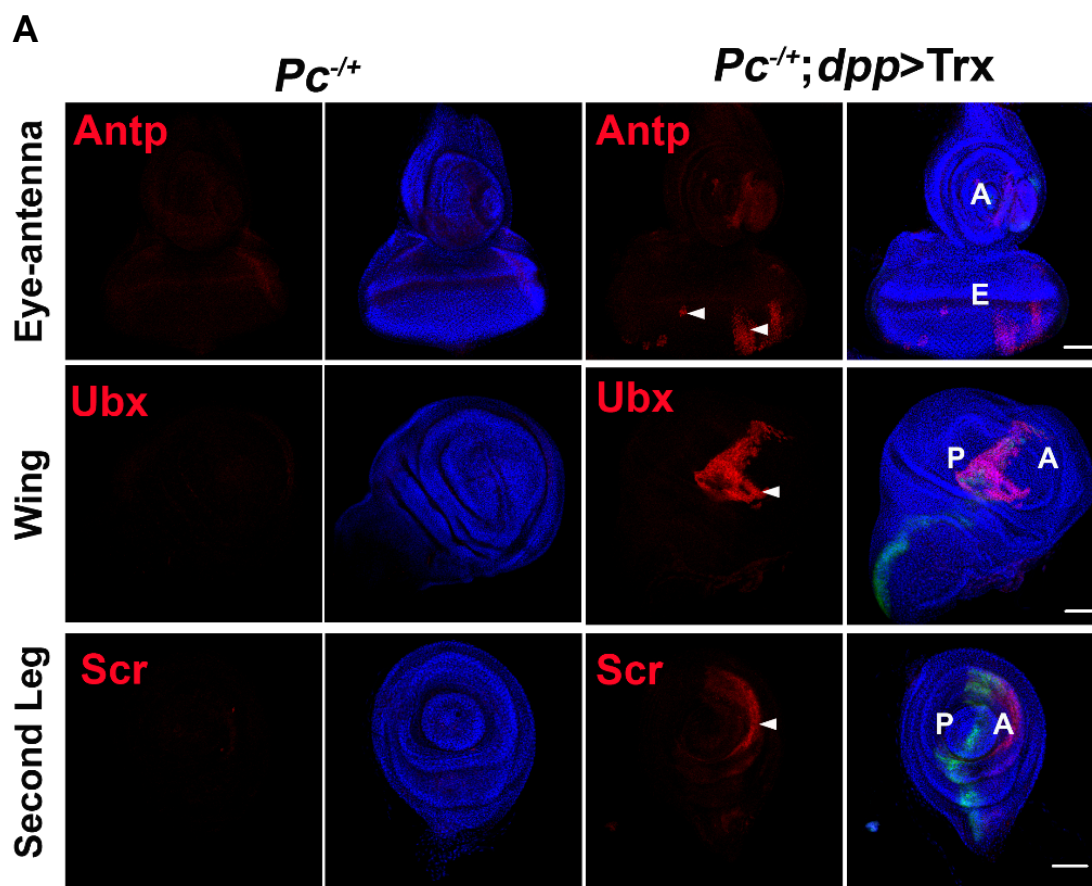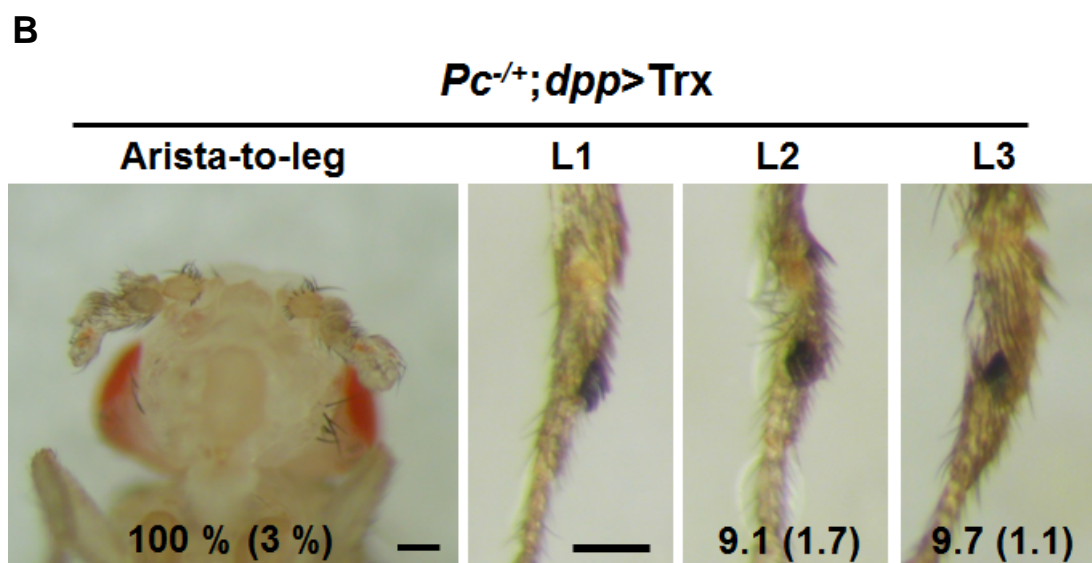

Supplement: S12 Fig — (A) Ectopic Hox expression in Pc4 heterozygote mutants. Antp, Ubx and Scr expression patterns in Pc-/+ (left) or Pc-/+;dpp>Trx (right) backgrounds are shown for eye-antenna, wing and L2 disc, respectively. Arrowheads indicate regions with deviant Hox expression in Fig 3A. For comparison, Hox expression induced by dpp>Trx alone is shown (Figs 2C, 2D and S6C). (B) Enhanced adult phenotypes in Pc4 heterozygote mutants. Arista-to-leg transformation is seen in all Pc-/+;dpp>Trx adults (left panel). The percentages of transformed dpp>Trx adults are indicated in parentheses. Strong transformation of L2/L3 is seen in Pc-/+;dpp>Trx adults (right panel). The average numbers of sex comb teeth in Pc-/+;dpp>Trx are indicated for L2 and L3. The corresponding numbers in Pc-/+ mutants are indicated in parentheses. Scale bar, 0.1 mm. (PDF) [file pgen.1005897.s012.pdf]
