# Supplementary material for: Maintenance of Tissue Pluripotency by Epigenetic Factors Acting at Multiple Levels
Source: PLoS Genet. 2016 Feb 29;12(2):e1005897. doi: 10.1371/journal.pgen.1005897 (PMC4771708; doi:10.1371/journal.pgen.1005897)
Supplement: S1 Table — (PDF) [file pgen.1005897.s013.pdf]

S1 Table. Wing phenotypes in RNAi backgrounds.

Function	*UAS-RNAi/ Mutant*	Gal4	Wing phenotype	Stock ID
**Control**	TRXi	*dpp*	+++	GD4502
**Negative Elongation Factors**	NELF-Di	*A9* & *dpp*	+++	GD42217
	NELF-EIRi	*A9* & *dpp*	++	BL6788
	NELF-Bi	*dpp*	+	GD17038
**Positive Elongation Factors**	PAFi	*dpp*	+++	GD20876
	CDK7i	*A9*	-	GD10442
	CYCTi	*dpp*	-	GD37562
	SPT6i	*dpp*	-	GD31703
	SPT5i	*dpp*	+	GD19793
	$Spt5^{MGE-3}$	*dpp*	+	BL8352*
**Chromatin Remodeling Factors and others**	ASFi	*dpp*	++	GD23737
	SPT16i	*dpp*	-	GD10916
	BRMi	*A9*	-	GD37720
	CHDi	*dpp*	-	GD26277
	NURFi	*A9*	-	GD24740
	ASH1i	*A9*	-	GD28982
	$ash1^{22}$	*A9*	-	BL24161*
	Nej^{P}	*A9*	+	BL3728*

Females carrying *UAS-Trx* with or without *UAS-RNAi*/*Mutant* were crossed to *dpp-Gal4* or *A9-Gal4* males, except for CDK7i, BRMi, ASH1i, NURFi, $ash1^{22}$ and Nej^{P}. In these cases, females carrying *A9-Gal4* and *UAS-RNAi*/*Mutant* were crossed to *UAS-Trx* males. Wing phenotype produced by *dpp-Gal4* is used as the reference. Progenies with similar phenotypes or restored wing are marked as (-) or (+), respectively. Fully restored wings are indicated as (+++). "*", mutants used for screening. BL and GD refer to Bloomington and Vienna stock centers, respectively.
