# Supplementary material for: Maintenance of Tissue Pluripotency by Epigenetic Factors Acting at Multiple Levels
Source: PLoS Genet. 2016 Feb 29;12(2):e1005897. doi: 10.1371/journal.pgen.1005897 (PMC4771708; doi:10.1371/journal.pgen.1005897)
Supplement: S2 Table — (PDF) [file pgen.1005897.s014.pdf]

S2 Table. Genetic interaction between Pc and histone chaperone or variant mutations.

Mutant	***Pc*$^-$/Bal**	***Pc*$^-$/mutant**
His3.3A^{2X1}	1.4 ± 0.1 (100)	1.8 ± 0.2 (98)
dom^{3}	2.5 ± 0.2 (100)	7.2 ± 0.2 (100)
dom^{9}	2.2 ± 0.1 (100)	4.2 ± 0.2 (104)
H2Av^{810}	1.8 ± 0.2 (98)	5.9 ± 0.2 (102)

Females carrying *Pc*^{4} mutation were crossed to histone variants or chaperones mutant males. The number of extra sex comb teeth (ESCT) on both L2 and L3 of their progenies were scored. The average and SEM of ESCT per leg are shown. Total number of legs examined are indicated in parentheses. Note that none of these histone chaperone or variant mutants show ESCT phenotype alone. Bal, Balancer.
