# Supplementary material for: Maintenance of Tissue Pluripotency by Epigenetic Factors Acting at Multiple Levels
Source: PLoS Genet. 2016 Feb 29;12(2):e1005897. doi: 10.1371/journal.pgen.1005897 (PMC4771708; doi:10.1371/journal.pgen.1005897)
Supplement: S3 Table — (PDF) [file pgen.1005897.s015.pdf]

S3 Table. Induction of arista-to-leg transformation.

Trx Induced	Arista-to-leg	
	***dpp*>Trx**	***Hira*$^{-/Y}$; *dpp*>Trx**
96 h	0.6 (168)	2.8 (112)
120 h	2.3 (128)	7.3 (110)
144 h	2.5 (122)	22.0 (91)
168 h	3.7 (134)	39.5 (86)

Crosses were done as described in Table 3. The percentage of arista-to-leg transformation is shown. The number of scored arista is indicated in parentheses.
