# Supplementary material for: Maintenance of Tissue Pluripotency by Epigenetic Factors Acting at Multiple Levels
Source: PLoS Genet. 2016 Feb 29;12(2):e1005897. doi: 10.1371/journal.pgen.1005897 (PMC4771708; doi:10.1371/journal.pgen.1005897)
Supplement: S4 Table — (PDF) [file pgen.1005897.s016.pdf]

S4 Table. List of primers.

Primer	Sequence 5' to 3'	Position to TSS	Reference
Antp-P1	CGTGGGCTAAGTCAACGGAA<br>AAGGAGTTCTCGCTGGGATT	0	
Antp-P1 +0.1K	AGAGCCCAGCCCCAAGATA<br>CAACAAAGCACTCGAGGACC	+0.1 kb	
Antp-P1 +1K	CAACGTTTTCTAGTCCCCCG<br>AAAGTTTGCCCGCAGTGG	+1 kb	
Antp-P2	AAGCAAAGTGCGAATCGAGC<br>TCACTGGCGTTCAGTTGTGA	0 kb	
Antp-P2 +0.1K	CTGGGAATGAACCGAACGGA<br>GACGTGCCAAATAGACGTGC	+0.1 kb	
Antp-P2 +1K	TGTGTGGAAACGATGCGAATG<br>AACGAGTGTAAAGCCGAGCA	+1 kb	
Antp-3'	CTCGCCCTTCGTCTTGTTCT<br>GAAGGCAGACATACACCCGG	+100.5 kb	
Scr-P1	ACTTCGCGGATTGCTACGC<br>AAGGCAACGCACGAGAGG	0 kb	
Scr-P2	ACACGCGGAACAGCTGAGT<br>GTATGAGTGAGCCAGAGCGCT	0 kb	
Scr-3'	CGAACTGCGACGGATGGATA<br>GAAGGAGCACAAGATGGCCT	+24 kb	
bxd-1	TAGTCTTATCTGTATCTCGCTCTTA<br>CAGAACCAAAGTGCCGATAACTC	-29.6 kb	[1]
bxd-2	GCACGCACTAAACCCCATAA<br>TCCACCTCCTCTTCCTCTCTC	-28.9 kb	
Ubx-P	GCGCTCTCTCTTGAGTGTTCGT<br>GGCGAGCGCATTTTCCTT	0 kb	[2]
Ubx +0.1K	TCCAATCCGTTGCCATCGAACGAAT<br>TTAGGCCGAGTCGAGTGAGTTGAGT	+0.1 kb	[1]
Ubx +1K	AATTGGTTTCCAGGGATCTGC<br>ATCCAAAGGAGGCAAAGGAAC	+1 kb	
bx	CCATAAGAAATGCCACTTTGC<br>CTCTCACTCTCTCACTGTGAT	+31.7 kb	
Ubx-3'	AGTTCCACACGAATCATTATCTGAC<br>CTTCATTCGCCGGTTCTGGA	+74 kb	

Primers used for RT-qPCR

Ubx	GCTCACTTCTACCAGACTGGC<br>CTTCATTCGCCGGTTCTGGA
Scr	CCGTGGATGAAGCGAGTACA<br>ACCCATGTGGTAGGGTACGA
Antp	ATGCCGTCTCCACTGTATCC<br>CGACGGGTCAAGTAGCGATT
Rpl32	ACTTCATCCGCCACCAGTCGG<br>CGCTCGACAATCTCCTTGCGC

References

1. Gambetta MC, Oktaba K, Muller J. Essential role of the glycosyltransferase sxc/Ogt in polycomb repression. Science. 2009;325(5936):93-6. doi: 10.1126/science.1169727 PMID: 19478141

2. Chopra VS, Hong JW, Levine M. Regulation of Hox gene activity by transcriptional elongation in Drosophila. Curr Biol. 2009;19(8):688-93. doi: 10.1016/j.cub.2009.02.055 PMID: 19345103
