# Supplementary material for: Maintenance of Tissue Pluripotency by Epigenetic Factors Acting at Multiple Levels
Source: PLoS Genet. 2016 Feb 29;12(2):e1005897. doi: 10.1371/journal.pgen.1005897 (PMC4771708; doi:10.1371/journal.pgen.1005897)
Supplement: S1 Text — (PDF) [file pgen.1005897.s017.pdf]

S1 Text. Chromatin immunoprecipitation assays (ChIP)

Third instar larvae from different genotypes were dissected in batch in M3 medium (Sigma-Aldrich) without serum for less than 20 min. After removal of guts and fat bodies, inverted carcasses with attached discs were fixed for 20 min at room temperature by gentle mixing for 5 min in 400 µl of 1% formaldehyde solution. After three changes of fixation solution, discs were washed at least 4 times for 2 min with 400 µl PBS containing 0.125 M glycine. Fixed carcasses were then washed 3 times for 5 min with 400 µl of PBT. Discs were separately collected. Pools containing 200~500 eye-antenna, wing, second leg or third leg discs were subjected to sonication in lysis buffer by a Bioruptor sonicator according to vendor's instructions (Diagenode). After sonication, insoluble materials were removed by centrifugation at 13000 rpm for 15 min in Microfuge. All samples were then stored at -80°C.

Chromatin aliquots were subjected to immunoprecipitation using manufacture's protocol (Millipore). Chromatin aliquots corresponding to 30-50 discs were diluted with dilution buffer (0.01% SDS, 1.1% Triton X-100, 1.2 mM EDTA, 16.7 mM Tris-HCl, pH 8.1, 167 mM Nacl, 1 mM PMSF) to 1 ml, followed by pre-clearing with 60 µl of protein-A magnetic beads (Invitrogene) for 30 min at 4°C. After the removal of beads, pre-cleared chromatin was immunoprecipitated by primary antibodies overnight at 4°C. Antibody-chromatin complexes were captured by incubation with 60 µl of protein-A magnetic beads for 2 h at 4°C. Recovered beads were washed with low salt, high salt and LiCl solution according to vendor's protocol for ChIP assay kit (Millipore). After two washes of TE solution, beads were eluted with 0.1M $NaHCO_3$ and 1% SDS, followed by reversal of cross-links. DNA was purified and quantified by qPCR in triplicate using SYBR Green mixture in standard settings (ABI 7500, Applied Bioscience). Data were collected from two ChIP experiments, each with two separate qPCR reactions. The primer

sequences are listed in S4 Table. Primary antibodies were mouse monoclonal antibodies against Ser5P (H14, Covance), Ser2P (H5, Covance) and control mouse IgM (Jackson ImmunoResearch). Secondary antibody was rabbit anti-mouse IgM (Jackson ImmunoResearch).
